# Spindle Position in Symmetric Cell Divisions during Epiboly Is Controlled by Opposing and Dynamic Apicobasal Forces

**DOI:** 10.1016/j.devcel.2012.01.002

**Published:** 2012-04-17

**Authors:** Sarah Woolner, Nancy Papalopulu

**Affiliations:** 1 Faculty of Life Sciences, Michael Smith Building, University of Manchester, Oxford Road, Manchester M13 9PT, UK

## Abstract

Orientation of cell division is a vital aspect of tissue morphogenesis and growth. Asymmetric divisions generate cell fate diversity and epithelial stratification, whereas symmetric divisions contribute to tissue growth, spreading, and elongation. Here, we describe a mechanism for positioning the spindle in symmetric cell divisions of an embryonic epithelium. We show that during the early stages of epiboly, spindles in the epithelium display dynamic behavior within the plane of the epithelium but are kept firmly within this plane to give a symmetric division. This dynamic stability relies on balancing counteracting forces: an apically directed force exerted by F-actin/myosin-2 via active cortical flow and a basally directed force mediated by microtubules and myosin-10. When both forces are disrupted, spindle orientation deviates from the epithelial plane, and epithelial surface is reduced. We propose that this dynamic mechanism maintains symmetric divisions while allowing the quick adjustment of division plane to facilitate even tissue spreading.

## Graphical abstract

**Graphical Abstract:**

**Figure:**
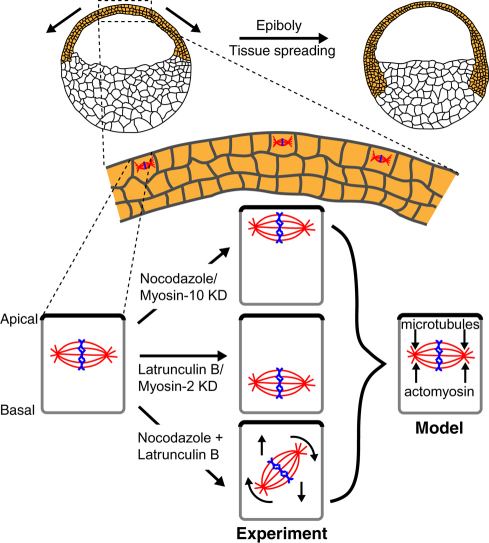

**Highlights:**

► Mitotic spindles show dynamic rotations but planar stability in symmetric divisions ► Spindle behavior in symmetric division is controlled by opposing apicobasal forces ► Microtubules and myosin-10 contribute a basally directed positioning force ► F-actin and myosin-2 provide an apically directed positioning force

## Teaser

Symmetric cell divisions are vital for tissue growth and depend upon the accurate positioning of the mitotic spindle. Woolner and Papalopulu show that during the symmetric divisions of a developing epithelium, spindles are held in place along the apicobasal axis through a balance of opposing, dynamic microtubule and actomyosin forces.

## Introduction

Epithelial tissues typically consist of apicobasally polarized cells, connected by specialized cell-cell junctions, often overlying one or more layers of basal nonpolar cells. Epithelial morphogenesis includes tissue spreading, folding, or stratification (thickening) of epithelial sheets and is an important aspect of embryogenesis, wound healing, and tissue engineering. Epithelial morphogenesis relies on a variety of cellular behaviors, for example, spindle orientation, cell shape changes, and cell intercalation. Among these, the control of mitotic spindle orientation occupies a key role in determining the outcome of cell division with respect to epithelial morphogenesis (Baena-López et al., 2005; da Silva and Vincent, 2007; Lechler and Fuchs, 2005). Spindles can be oriented either parallel or perpendicular to the plane of the epithelium. Parallel orientation underlies cell divisions that are symmetric and contribute to tissue spreading or elongation (Baena-López et al., 2005; da Silva and Vincent, 2007; Fleming et al., 2007). Perpendicular spindle orientation leads to asymmetric division and contributes to tissue thickening (Lechler and Fuchs, 2005; Poulson and Lechler, 2010).

Much of our knowledge of the mechanisms that control spindle orientation comes from studies of asymmetric division in invertebrate embryos, where spindles are thought to be positioned through forces generated by interactions between spindle astral microtubules and the cell cortex (Grill and Hyman, 2005; Marthiens et al., 2010; Siller and Doe, 2009). For example, in the first division of the *C*. *elegans* embryo, microtubule motors at the cell cortex pull on the astral microtubules of the spindle to position it closer to the posterior end of the cell, resulting in an asymmetric division with a smaller posterior blastomere (Grill et al., 2003; Kozlowski et al., 2007). In the *Drosophila* neuroblast, asymmetric division requires the apical localization of a complex of spindle orientation proteins, including Mud and Pins (Siller et al., 2006). This complex is then thought to recruit the microtubule motor, dynein, to the apical cortex, providing a pulling force that draws one spindle pole toward the apical surface (Siller and Doe, 2008, 2009). In the mouse epidermis, a similar complex of spindle orientation proteins localizes at the apical cortex to drive the asymmetric divisions that lead to the stratification and differentiation of the skin (Lechler and Fuchs, 2005; Poulson and Lechler, 2010).

In contrast to asymmetric division, we know much less about the mechanisms that position the spindle during symmetric cell divisions. Although it is known that cell adhesions are required to achieve a symmetric division (den Elzen et al., 2009; Lu et al., 2001; Toyoshima and Nishida, 2007), we have little idea about the nature of the forces that act to hold the spindle in place during these divisions. This is particularly true during morphogenesis, when spindle orientation needs to be dynamically linked to tissue-shaping events. In this case, it is not clear how different levels of positional control—allowing spindles to be held level to give a symmetric division while maintaining the freedom to adjust the direction of division within this plane—are mechanistically reconciled.

Here, we used the epithelium of the early gastrula *Xenopus* embryo as a model system to study the mechanisms of spindle orientation during symmetric cell divisions. At this stage the embryo is just beginning epiboly, a morphogenetic movement where the epithelium must spread in all directions to cover the embryo. We report that spindles are maintained within the plane of the epithelium (z axis) throughout mitosis but exhibit very dynamic rotations within this plane. These rotations continue until the onset of anaphase, when spindles are stabilized in variable directions, but always within the epithelial plane. We report that the spindle is maintained in the plane of the epithelium by the activity of two molecularly distinct sets of forces: a basally directed force, based on microtubules/myosin-10 (Myo10); and an apically directed force exerted by F-actin/myosin-2. We show that disruption of either of these forces repositions the spindle along the apicobasal axis, whereas disruption of both results in failure to maintain the spindle within the plane of the epithelium. This mechanism differs from previously described models of spindle positioning in that it relies on a dynamic balance of apicobasal forces rather than on tethering of the spindle to particular location(s) in the cortex (Marthiens et al., 2010). We suggest that this dynamic mechanism endows the spindle with vital flexibility to move and when needed, rapidly adjusts its orientation within the plane of the epithelium, while maintaining the overall process of symmetric divisions.

## Results

### Mitotic Spindles in Embryonic Epithelia Show Dynamic Rotations but Planar Stability in Symmetric Division

**Figure 1:**
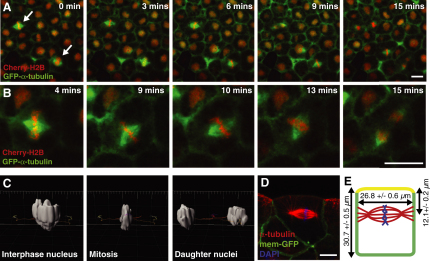
Mitotic Spindle Position in a Developing Epithelium (A) Stills taken from a single focal plane movie of mitotic spindles in the outer epithelial layer of a *Xenopus laevis* embryo. Embryos were injected with GFP-α-tubulin (green) to label spindles and Cherry-histone2B (Cherry-H2B) to highlight chromosomes. Spindles are aligned parallel to the plane of the epithelium but are also held in a specific position along the apicobasal axis of the cell; spindles in neighboring cells assemble in the same focal plane and remain here throughout mitosis (arrows). (B) Zoom-in of movie in (A) shows that spindles undergo rapid rotational movement in the x/y plane, while remaining held in parallel orientation and apicobasal position. (C) 3D reconstruction of Cherry-H2B fluorescence from a z stack confocal movie (Movie S2), which can be used to track the apicobasal position of nuclei as mitosis proceeds (side view; apical at top, basal at bottom). Virtually no movements in the apicobasal axis are seen. (D) A side-view image of a mitotic spindle in the outer epithelium of a fixed embryo (apical at top, basal at bottom) demonstrates the apicobasal position of spindles in these cells. (E) Mean values for cell length, width, and distance of spindle from apical surface are shown (±SEM, n = 91 spindles in 18 embryos). Scale bars represent 20 μm in (A) and (B) and 10 μm in (D). See also Movies S1 and S2.

To investigate the forces acting to position the spindle in symmetrically dividing cells, we used early *Xenopus laevis* embryos, which provide a powerful system to study spindle dynamics in vivo in a whole organism (Kieserman et al., 2010; Woolner et al., 2009). We concentrated on divisions in the outer epithelial cell layer of the early gastrula (stage 10–10.5), where spindles can be readily imaged using live confocal microscopy (Woolner et al., 2009) (Figure 1A; see also Movie S1 available online). These cells exhibit typical epithelial apicobasal polarity (Chalmers et al., 2003, 2005), but they differ from mature epithelia in that they lack a basal lamina (Marsden and DeSimone, 2001) and do not round up in mitosis. At this stage in development, embryos are undergoing epiboly, a morphogenetic process where the cells of the animal cap spread over the embryo. As reported previously (Tabler et al., 2010; Woolner et al., 2008), we find that the majority of spindles (84% ± 8.4%) in this developing epithelium align parallel to the plane of the epithelium and undergo symmetric divisions (Figure 1A; Movie S1). This orientation is established as the spindle assembles and is maintained throughout mitosis. However, although spindles maintain a parallel orientation, they undergo rapid movements in the x/y (planar) axis, rotating throughout metaphase and only stopping once anaphase has begun (Figure 1B; Movie S1) (Woolner et al., 2008). In single focal plane (single z) movies (Figures 1A and 1B), we also noticed that spindles assembled in the same focal plane (all in focus) and remained in this z position throughout mitosis. This indicates that a third level of positional control is acting on these spindles, positioning them along the apicobasal axis of the cell. To study this position in greater detail, we reconstructed z stack movies of the nuclei of dividing cells (Figure 1C; Movie S2) and tracked nuclei position through division. We saw very little movement of the condensed chromosomes in the z axis, indicating that spindles are held stably along the apicobasal axis of the epithelial cells. Indeed, quantification of the apicobasal position of spindles in fixed cells (Figure 1D) showed a tight distribution, indicating that position is not random but fixed and biased toward the apical surface (Figures 1E and 4C).

The spindles in this embryonic epithelium are therefore under three levels of positional control: parallel orientation, apicobasal position, and planar rotation. However, the dynamics of these controls are different, with parallel orientation and apicobasal position being established and maintained from the start of mitosis, whereas planar rotations proceed throughout metaphase and only stop once anaphase begins. To understand the mechanism that endows the spindles with stability in maintaining a planar direction of division coupled with dynamic behavior in orienting within the plane (dynamic stability), we sought to identify the molecular forces exerted on the spindle.

### Spindles Are Not Aligned to Cell-Cell Junctions

**Figure 2:**
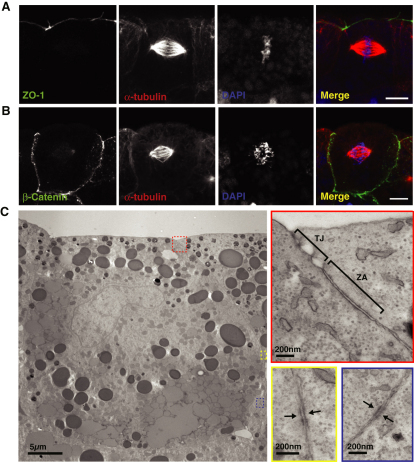
Spindle Position Does Not Correspond to Cell-Cell Junction Location (A) Immunofluorescence for ZO-1 (green), a component of tight junctions, shows that spindle position does not correlate with the location of tight junctions. (B) β-Catenin (green), a component of adherens junctions, is localized all around the basolateral cell surfaces. (C) Transmission electron micrographs (zoomed-in areas highlighted in red, yellow, and blue boxes) show that tight junctions (TJ) and the zona adherens (ZA) are located apically and stretch no more than 2.5 μm down from the apical cell membrane (red box), whereas regions of high density, which may be cell-cell contacts (yellow and blue boxes; arrows), are found at random positions around the basolateral membranes. Scale bars represent 10 μm in (A) and (B) and are as displayed in (C).

Previous findings have shown that adherens junctions are required to maintain the spindle in a parallel orientation during symmetrical divisions in *Drosophila* and mammalian epithelial cells (den Elzen et al., 2009; Lu et al., 2001). This suggests a simple model whereby spindles align to the cortical position of cell-cell junctions, perhaps guided by centrosome capture and microtubule-based pulling forces. Following on from this model, one would expect that cell-cell junctions would be found in approximately the same z position as spindles (Marthiens et al., 2010). However, using both immunofluorescence and transmission electron microscopy (TEM), we were unable to find a junctional structure that correlated with spindle position. Tight junctions were found in a much more apical position compared to the spindle (Figures 2A and 2C). Components of the adherens junction were spread around the basolateral surfaces (Figure 2B; β-catenin is shown here, and C-cadherin shows a similar localization). TEM showed that whereas the zona adherens had a specific location, this was only just basal to the tight junction and so, when estimated by distance from the apical surface, was not aligned to spindle position (Figure 2C). By TEM we also found regions of high density, which may correspond to cell-cell contacts; however, these were found at several positions around the basolateral surface (Figure 2C). The lack of alignment to any specific type of cell-cell junction argues against a simple model of spindle positioning in which the spindle tethers level with the junction. These findings do not rule out a role for cell-cell junctions in spindle position but suggest that additional mechanisms are required to determine the exact location of the spindle.

### Disruption of Astral Microtubules Causes Spindles to Move Apically

**Figure 3:**
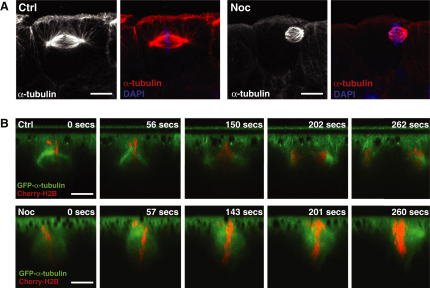
Treatment with Low-Dose Noc Specifically Disrupts Astral Microtubules and Causes Spindles to Reposition Apically (A) Spindles in control (Ctrl) versus Noc-treated embryos; treatment with Noc eradicates astral microtubules that are seen in Ctrl spindles and causes spindles to move to the apical cell surface. (B) Stills taken from Movie S3, showing spindles in Ctrl and Noc-treated embryos. The Noc-treated spindle moves toward the apical cell surface, whereas the Ctrl spindle remains in a constant position along the apicobasal axis even as anaphase proceeds. Scale bars represent 10 μm. See also Figure S1 and Movies S3 and S4.

**Figure 4:**
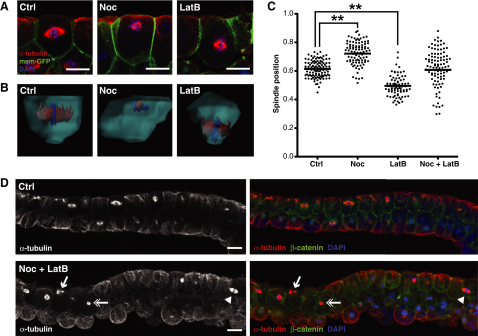
Disruption of Astral Microtubules and Actin Filaments Has Contrary Effects on Spindle Position (A) Side-view immunofluorescent images of spindles in control (Ctrl), Noc, and LatB-treated embryos. Treatment with low-dose Noc causes spindles to move toward the apical cell surface, whereas spindles in LatB-treated embryos move toward the basal surface. (B) 3D reconstructions of individual cells from the epithelium of Ctrl, Noc, and LatB embryos. (C) Quantification of spindle position in Ctrl, Noc, LatB, and Noc + LatB-treated embryos; each dot represents the position of a single spindle. Note that in double-treated embryos the spread of spindle position data are much greater than in either single treatments or control. For significance testing, unpaired Student's t tests were performed (n = 3 independent experiments, from a total of 19, 18, 16, and 16 embryos for Ctrl, Noc, LatB, and Noc + LatB, respectively; ^∗∗^p < 0.01). (D) Immunofluorescence images of Ctrl and Noc + LatB-treated embryos. In double-treated embryos, spindles are seen randomly positioned along the apicobasal axis, with spindles seen at the apical surface (arrow), basal surface (arrowhead), and center of the cell (double arrow). Scale bars represent 20 μm. See also Figure S2.

Astral microtubules are key structures in anchoring and positioning the spindle, so we sought their role in these symmetric divisions. We reasoned that if astral microtubules simply anchor spindles to a particular cortical location, we would expect disruption of astral microtubules to randomize spindle position. To test this model, we specifically disrupted astral microtubules by treating embryos with a low concentration of nocodazole (Noc) for 30 min, which inhibited astral microtubules but left the spindle intact (Figure 3A). However, instead of randomizing spindle position, we found that Noc treatment caused spindles to consistently reposition closer to the apical cell surface (Figures 3A and 4A–4C; Noc spindles were positioned 31% closer to the apical surface than control spindles). Live imaging of Noc-treated embryos revealed that spindles moved to the apical surface during mitosis and that their repositioning was not just a consequence of mislocalized interphase nuclei (Figure 3B; Movie S3).

In other systems, notably in yeast and *C*. *elegans*, astral microtubules position the spindle by exerting pushing or pulling forces, depending on the system, associated with microtubule polymerization and depolymerization, respectively. Both models involve molecular motors and microtubule contact with cortical sites (Dogterom et al., 2005; Grill and Hyman, 2005; Nguyen-Ngoc et al., 2007). To gain a better understanding of any microtubule-based forces in our system, we subjected embryos to increasing concentrations of Noc, or increasing exposure times to Noc. First, we found that as Noc concentration increased, spindles became more apically positioned (Figures S1A and S1B). At very high concentrations, where there were very few, if any, spindle microtubules, condensed chromosomes were found pressed against the apical cell surface (Figure S1A). Because microtubule density decreases with increasing doses of Noc, the direction of movement is consistent with a reduction of microtubule-based pushing forces. However, Noc treatment may also increase the depolymerization rate of microtubules in contact with the cortex and hence increase forces that may pull the spindle apically. Therefore, this experiment alone does not rule out the presence of pulling forces.

To clarify this, we next used increased time exposure to a constant concentration of Noc, chosen to allow spindle assembly (Movie S4) but disrupt astral microtubules (Figure S1A). We reasoned that with increased time of incubation in Noc, the number of spindles that assemble in the presence of Noc would increase, and hence, the number of astral microtubules that make contact with the apical cortex would decrease. If there are pulling forces exerted by depolymerization of microtubules as they contact the apical cortex, one would see a decrease in the number of spindles that move apically with increasing Noc incubation. However, this is not what we found; we saw no reduction in apically positioned spindles in longer Noc incubations compared to shorter incubations (Figure S1C). The simplest interpretation of these two experiments together is that the presence of microtubules restricts the apical position of the spindle, either by acting as a barrier or by exerting a basally directed pushing force.

### F-Actin Provides an Opposing Force to Position the Spindle

If microtubules resist the apical-ward movement of the spindle, then what is the source of the antagonistic apically directed force that balances spindle position? Antagonistic relationships between the microtubule and actin cytoskeletons have been described elsewhere in biology (Mandato et al., 2000); therefore, we asked whether F-actin was actively involved in apicobasal spindle position. We treated embryos with Latrunculin B (LatB) to disrupt actin filaments and then measured apicobasal spindle position. We found that disruption of F-actin had the opposite effect on spindle position to Noc treatment: spindles now moved toward the basal cell surface (Figures 4A–4C). This result suggests that, normally, a balance of actin and microtubule-based forces positions the spindle, with F-actin providing an apically directed force and astral microtubules providing a basally directed force. We reasoned that if this balance of cytoskeletal forces was the main driver of apicobasal spindle position, then removing both F-actin and astral microtubules should randomize spindle position. We found that this was indeed the case because treatment with both Noc and LatB caused spindles to be positioned randomly along the apicobasal axis, as can be seen by immunofluorescence (Figure 4D) and confirmed by the wide spread of spindle position measurements seen in double-treated embryos (Figure 4C; control SD = 0.06, Noc + LatB SD = 0.12). This force-balancing mechanism appears to operate only during mitosis because we did not see the same effects on nuclei positioning in interphase cells (Figure S2A).

### Myo10 Functions Antagonistically to F-Actin to Position the Spindle

**Figure 5:**
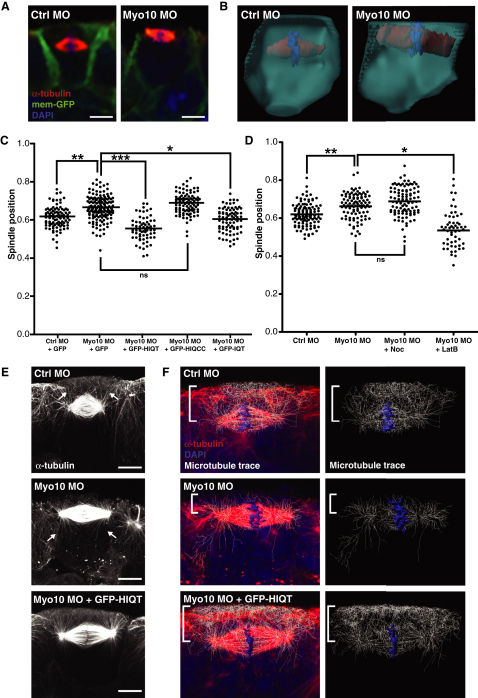
Myo10 Helps Position the Spindle but Functions Antagonistically to F-Actin (A) Side-view immunofluorescent images of spindles in control morpholino (Ctrl MO) and Myo10 MO-injected embryos. (B) 3D reconstructions of single cells from epithelium of Ctrl MO and Myo10 MO embryos. (C) Quantification of spindle position in Ctrl MO, Myo10 MO (both coinjected with GFP as a control), and Myo10 MO rescued with full-length GFP-tagged Myo10 (GFP-HIQT), tailless Myo10 (GFP-HIQCC), or headless Myo10 (GFP-IQT). Spindles are repositioned closer to the apical cell surface in Myo10 MO embryos compared to Ctrl MO, a phenotype rescued by coinjection with full-length or headless Myo10, but not tailless Myo10. To test for significance, unpaired Student's t tests were performed (n = 5 independent experiments for Ctrl MO + GFP and Myo10 MO + GFP, from a total of 27 and 32 embryos, respectively; n = 3 independent experiments, from a total of 18 embryos each for Myo10 MO + GFP-HIQT, + GFP-HIQCC, and + GFP-IQT; ^∗^p < 0.05, ^∗∗^p < 0.01, ^∗∗∗^p < 0.01). (D) Quantification of spindle position in Ctrl MO, Myo10 MO (both treated with 0.1% DMSO as a control), and Myo10 embryos treated with Noc or LatB. Treatment with low-dose Noc does not affect Myo10 MO spindle position, but LatB treatment of Myo10 MO embryos causes spindles, on average, to move basally and results in a wider spread of spindle position. To test for significance, unpaired Student's t tests were performed (n = 3 independent experiments, from a total of 20, 16, 18, and 11 embryos for Ctrl MO, Myo10 MO, Myo10 MO + Noc, and Myo10 MO + LatB, respectively; ^∗^p < 0.05, ^∗∗^p < 0.01). (E) High-resolution side-view confocal images (stacks of 13 z slices for each condition) of spindle microtubules in Ctrl MO, Myo10 MO, and Myo10 MO rescued with GFP-HIQT. In Ctrl MO cells, spindle microtubules have an apical asymmetry, with more astral microtubules on the apical side (arrows). Spindles in Myo10 MO cells lose this asymmetry, and long basal astral microtubules are seen (arrows). The GFP-HIQT rescue restores the apical asymmetry. (F) Filament tracing of the microtubule signal (white trace of red staining) provides an unbiased approach to view the asymmetry of the microtubule network. For each image, the trace represents only microtubules present in the central mitotic cell: any traces originating in neighboring cells were deleted. In particular, a dense network of microtubules is seen on the apical side of Ctrl MO spindles, which is lost in the Myo10 MO and restored in the GFP-HIQT rescue (square bracket). ns, not significant. Scale bars represent 10 μm. See also Figure S3.

To unravel the molecular forces involved in spindle positioning, first we looked at Myo10 because this unconventional myosin has been shown to be involved in spindle orientation in cultured cells (Toyoshima and Nishida, 2007) and spindle rotation in embryonic epithelium (Woolner et al., 2008). In addition, Myo10 can bind directly to microtubules (Weber et al., 2004), as well as F-actin, opening up the possibility that Myo10 may be able to provide a link between astral microtubules and cortical F-actin. To investigate a possible function for Myo10 in apicobasal spindle positioning, we knocked down Myo10 protein levels by microinjecting embryos with an antisense morpholino oligo targeted to Myo10 (Myo10 MO), as described previously (Woolner et al., 2008). We found that knockdown of Myo10 led spindles to position much closer to the apical cell surface when compared to embryos injected with a standard control morpholino (Figures 5A–5C). As with Noc and LatB treatment, nuclei position in interphase cells was not affected by Myo10 knockdown (Figure S3A). The apical spindle position phenotype seen in morphants could be rescued by expression of full-length Myo10 (GFP-HIQT; Figure 5C). The localization of Myo10 is consistent with a role in spindle positioning because it localizes to both the spindle and the cell cortex (Figure S3B).

The mispositioning of the spindle at the apical surface in Myo10 morphants was opposite to disrupting F-actin, suggesting that Myo10 may function antagonistically to actin. Indeed, simultaneous disruption of F-actin, using LatB, and Myo10 using morpholino knockdown led to a greater spread of spindle position (Figure 5D). This was similar to the randomization effect seen when wild-type embryos were treated with both Noc and LatB (Figure 4C). In contrast, the effect of treating Myo10 morphants with Noc was not significantly different from the Myo10 MO alone (Figure 5D).

To investigate which domains of Myo10 are important for its function in spindle positioning, we performed rescue experiments using two previously described Myo10 truncations (Weber et al., 2004; Woolner et al., 2008): one that lacks the microtubule-binding tail but retains the actin-binding head domain (GFP-HIQCC), and one that lacks the actin-binding head but retains the microtubule-binding tail (GFP-IQT). We found that the microtubule-binding GFP-IQT construct could rescue the positioning defect seen in the Myo10 morphant, whereas the actin-binding, GFP-HIQCC, construct could not (Figure 5C). In a control background, we found that both GFP-IQT and the full-length, GFP-HIQT, construct caused spindles to reposition slightly basal, whereas the GFP-HIQCC construct mimicked the Myo10 MO phenotype, causing spindles to move apically, suggesting that it has a mild dominant-negative effect (Figure S3C). There are two possible ways to explain why the GFP-IQT construct causes spindles to move basally: either it is agonizing the microtubule-dependent force or antagonizing the actin-dependent force. We believe that the former explanation is correct because the expression of GFP-IQT rescued the Myo10 morphant with a tight distribution of spindle positioning, instead of randomizing spindle position as is seen when morphants are treated with LatB (Figures 5C and 5D). We, therefore, conclude that the microtubule-binding tail is required but that the actin-binding head is dispensable for Myo10's function in spindle positioning.

Because Myo10 appeared to be functioning agonistically with microtubules, we investigated if knockdown of Myo10 affected microtubule organization. As described previously (Woolner et al., 2008), we found that spindles in the Myo10 morphants were longer than controls (Figure S3D), but we also noticed a difference in the distribution of microtubules. In control spindles, astral microtubules showed an apical bias in their density, with many more microtubules reaching to the apical surface compared to the basal (Figure 5E). These astral microtubules contributed to a dense microtubule network seen across the apical surface of control cells (Figure 5F). In contrast, spindles in Myo10 morphants showed a more symmetrical distribution, characterized by a reduction in the dense apical microtubule network and an increased number of basal microtubules (Figures 5E and 5F). This dense network may provide an “apical barrier,” preventing the spindle from positioning more apically in control cells. This barrier appears dynamic, and Myo10 may function in its regulation, assembly, or maintenance. Indeed, we found that rescuing the Myo10 morphant phenotype with full-length Myo10 coincided with a complete restoration of the apical microtubule network (Figures 5E and 5F).

### Spindle Positioning Requires Myosin-2

**Figure 6:**
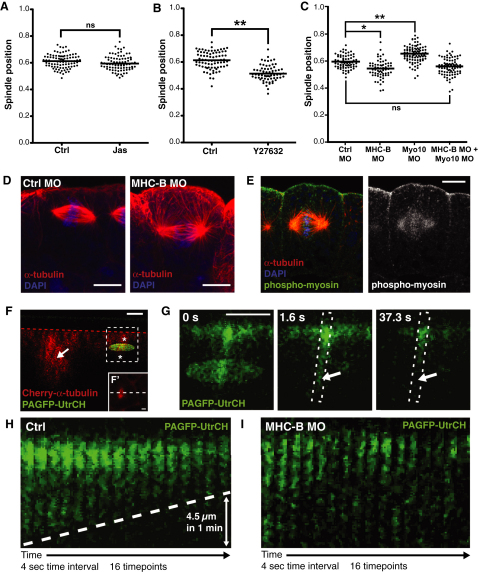
Myosin-2 Functions with F-Actin to Position the Spindle (A) Quantification of spindle position in control (Ctrl) and Jas-treated embryos indicates no effect on spindle position when F-actin turnover is disrupted. ns, not significant. (B) Inhibition of myosin-2 by the Rho kinase inhibitor, Y27632, causes spindles to position closer to the basal surface, compared to controls (^∗∗^p < 0.01). (C) Knockdown of myosin-2 function using a morpholino against myosin heavy-chain B (MHC-B MO) also causes spindles to position more basally compared to controls (Ctrl MO). Double knockdown of myosin-2 and Myo10 rescues the effects seen in single knockdowns, and spindles are positioned as in controls. To test for significance, unpaired Student's t tests were performed (in B, n = 4 independent experiments, from a total of 20 and 30 embryos for Ctrl and Y27632, respectively; in C, n = 3 independent experiments, from a total of 21 embryos for Ctrl MO, MHC-MO, and Myo10 MO, and 20 embryos for MHC-B MO + Myo10 MO; ^∗^p < 0.05, ^∗∗^p < 0.01). ns, not significant. (D) Confocal images of spindles in Ctrl MO and MHC-B MO embryos; note the basal position of the MHC-B MO spindle. (E) Staining for active myosin-2 (phospho-myosin; green) shows a strong accumulation apically, trailing off on the lateral sides in a basal direction. (F) To test for an apically directed cortical flow of actin filaments, photoactivatable-GFP fused to the GFP-UtrCH probe (PAGFP-UtrCH) was used (green). Cherry-α-tubulin (red) was coexpressed to identify mitotic cells, which were imaged in z (F′ shows an x/y image of the cell in F; white dashed line indicates the line of cross-section for F), and a zone of photoactivation (green oval) was positioned across the cell cortex (asterisks) approximately 5 μm down from apical cell surface (indicated by red dashed line). (G) A zoom-in of the region indicated by dashed box in (F); photoactivation causes an accumulation of fluorescence apical of the zone of photoactivation. To assess any movement of F-actin, we followed the loss of fluorescence that occurs once photoactivation is stopped. We saw progressive loss from basal to apical (arrows) over time, suggesting an apical-ward movement of F-actin. (H) Kymographs (of boxed region in G) show the progressive loss of fluorescence from basal to apical, the gradient of which (dashed line) can be used to estimate the speed of F-actin movement. In this case, the fluorescence front receded apically by 4.5 μm in 1 min. (I) Knockdown of myosin-2 function (MHC-B MO) stops the directional loss of fluorescence, indicating stalled F-actin movement. Four control embryos and three MHC-MO injected embryos were analyzed, with similar results. Scale bars represent 10 μm in (D) and (E) and 5μm in (F), (F′), and (G); time stamps indicate time in seconds. See also Figure S4 and Movies S5 and S6.

Because Myo10 did not facilitate the apically directed force provided by F-actin, we investigated two other likely alternatives: first, that an apically directed force could be generated by the turnover of F-actin; or second, that force could be generated by actomyosin contraction. To test these two possible mechanisms, we treated embryos with either jasplakinolide (Jas), which disrupts actin turnover by stabilizing actin filaments (Bubb et al., 1994; Cramer, 1999), or with Y27632, a Rho kinase inhibitor that indirectly inhibits myosin-2 (Davies et al., 2000). We found no effect on spindle position when F-actin was stabilized following treatment with Jas (Figure 6A), suggesting that dynamic turnover is not essential for actin's role in spindle positioning.

However, treatment with Y27632 caused spindles to move toward the basal cell surface, similar to LatB treatment (Figure 6B). To verify that this result was caused by inhibition of myosin-2, we tested directly whether morpholino knockdown of myosin-2 would cause a similar mispositioning of the spindle. Vertebrates have three myosin-2 heavy-chain isoforms (MHC-A, MHC-B, and MHC-C), and we chose to knock down MHC-B using a previously described morpholino (Skoglund et al., 2008). This isoform was the best functional candidate for gastrula stage embryos because it has been previously shown to be required for the completion of gastrulation. We found that MHC-B MO-injected embryos showed a similar basal mispositioning of the spindle to that seen with Y27632 (Figures 6C and 6D). As with LatB treatment, we saw no change in the position of interphase nuclei in MHC-B morphants (Figure S4A). In addition, we found that simultaneous knockdown of MHC-B and Myo10 suppressed the spindle mispositioning seen in either of the single knockdowns, indicating that these myosins function antagonistically to position the spindle (Figure 6C). We next investigated whether spindle structure was affected in MHC-B morphants. We found no significant effect on spindle length (Figure S4B), but we did see an expansion of the apical microtubule network (Figure S4C). This expansion again argues that the barrier is dynamic and is consistent with the idea that the apical microtubule network restricts the apical position of spindle because it corresponds to spindles positioning more basally in the MHC-B morphants.

### Spindles Are Linked to an Apically Directed Cortical Flow

Together, the Y27632 and MHC-B MO results indicate that myosin-2 functions agonistically to F-actin, and antagonistically to microtubules and Myo10, to position the spindle. An apical barrier formed by microtubules could explain why spindles are excluded from the apical portion of the cell but does not explain why spindles move apically when this barrier is removed. However, as we have shown, a loss of either F-actin (LatB treatment) or myosin-2 (MHC-B MO) prevents this apical repositioning, suggesting that actomyosin contraction may be moving spindles apically. To investigate this model further, we first determined the localization of active myosin-2 (visualized using an antibody against serine-19 phosphorylated myosin light chain) and found it concentrated at the apical surface, with a gradient of localization increasing from basal to apical (Figure 6E). We can exclude that the lack of basal staining is due to incomplete antibody penetration because of the basolateral β-catenin staining achieved using identical methods (Figure 2B). The graded localization of phospho-myosin would be consistent with apical actomyosin contraction providing an apically directed force to position the spindle, as occurs during oocyte spindle positioning (Schuh and Ellenberg, 2008). However, asymmetries in actomyosin contraction have also been shown to generate cortical flows of actin filaments from regions of relaxation to regions of contraction, which would predict a basal-to-apical flow in these cells (Bray and White, 1988; Hird and White, 1993; Munro et al., 2004). Because myosin-2 based cortical flow has been shown to be involved in centrosome separation during mitosis (Rosenblatt et al., 2004) and has been postulated previously in epithelial cells (Jacobson et al., 1986), we tested whether cortical flow could provide an apically directed force in this system.

To directly assess the movement of actin filaments in these cells, we used a construct that combines photoactivatable-GFP (Patterson and Lippincott-Schwartz, 2002) with the Utr-CH F-actin probe (PA-GFP-UtrCH) (Burkel et al., 2007). Using this probe, we photoactivated a small region of the cell cortex of a mitotic cell and allowed the cortex to become saturated with fluorescence (Figures 6F and 6G). We then turned off the photoactivation laser and assessed the direction of any F-actin flow by following the movement of GFP fluorescence and its replacement with nonactivated PA-GFP-UtrCH (Figure 6G; Movie S5). In this way, we found that fluorescence loss spread from basal to apical, indicating that F-actin was moving in an apical direction along the cell cortex (Figure 6G; Movie S5). Using kymographs (Figure 6H; Movie S6), we estimated the rate of F-actin flow in control cells to be 5.9 ± 0.7 μm/min (n = 4 embryos), a similar rate to that described for cortical flow in other systems (Canman and Bement, 1997; Hird and White, 1993). We then tested whether myosin-2 was required for this cortical flow and found that it was: in MHC-B morphants there was no directional movement of actin filaments (Figure 6I; Movie S6). Thus, our results are consistent with a model whereby a gradient of myosin-2 activity, and therefore contraction, instigates a cortical flow of actin filaments from regions of greater relaxation (basal) to regions of greater contraction (apical). The spindle could then be linked to this flow and carried apically.

### Opposing Microtubule and F-Actin Forces Keep Spindles Level to Ensure Symmetric Cell Division

**Figure 7:**
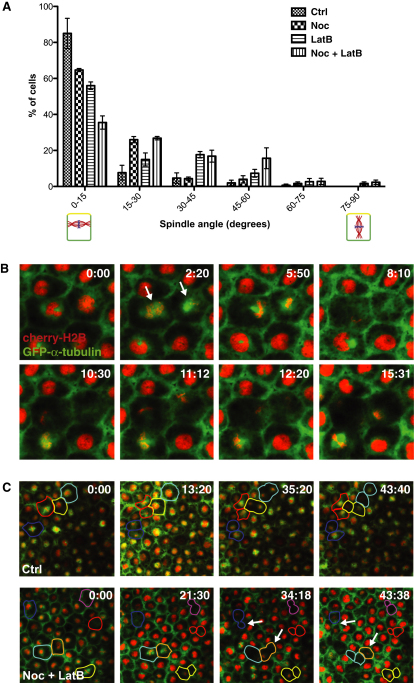
Microtubules and F-Actin Show Partial Redundancy in Spindle Orientation (A) Quantification of spindle angle in Ctrl, Noc, LatB, and Noc + LatB-treated embryos. Spindle angle was measured relative to the x/y plane, such that a spindle angle of 0° denotes a spindle that is oriented parallel to the epithelium and will undergo a symmetric division, and 90° denotes a spindle that is oriented perpendicular and will undergo an asymmetric division. Treatment with either Noc or LatB alone causes a slight reduction in parallel spindles, but a much larger reduction is seen in double-treated, Noc + LatB, embryos. Error bars represent SEM (n = 3 independent experiments, from a total of 17, 16, 19, and 20 embryos for Ctrl, Noc, LatB, and Noc + LatB, respectively). (B) Stills from Movie S7, following two spindles (arrows) in a Noc + LatB-treated embryo. Both spindles undergo random rotations out of the plane of the epithelium. (C) Cell perimeters in Ctrl and Noc + LatB embryos traced through one cell division. Each Ctrl division results in two daughter cells of similar apical surface area; divisions in Noc + LatB produce daughter cells of differing apical cell surface. In some cases (arrows) cells with a smaller apical surface are lost from the epithelial layer. See also Figure S5 and Movie S7.

Our findings indicate that spindles in this embryonic epithelium are positioned by balancing counteracting microtubule and actomyosin forces. We speculated that this same balance of forces could be used to keep the spindle poles level in order to give a symmetric division. To investigate the relative effects of disruption of astral microtubules and F-actin on spindle orientation, we measured spindle angle (relative to the x/y axis) in control and drug-treated embryos. We found that whereas single treatments with Noc or LatB caused a slight loss of parallel (0°–15°) spindles (Figure 7A), a much greater loss of parallel orientation was seen with the double, Noc + LatB, treatment. This indicates a functional redundancy between astral microtubules and F-actin in spindle orientation and shows that, just like apicobasal positioning, parallel orientation requires an active contribution from both the microtubule and actin cytoskeletons.

Live imaging of spindles in double-treated, Noc + LatB, embryos revealed that loss of parallel orientation resulted from “tumbling” of the spindles in and out of the plane of the epithelium (Figure 7B; Movie S7; note how parts of the spindles appear and then disappear). This is in great contrast to control spindles, which maintain their parallel orientation throughout mitosis and only undergo rotations strictly in the plane of the epithelial layer (Figures 1A and 1B; Movie S1; the whole spindle is in focus for the duration of mitosis). To assess the consequence to the epithelium of these “tumbling” spindles, we tracked cell divisions in control and double-treated embryos (Figure 7C). Although control divisions gave two daughter cells with equal apical cell surfaces, divisions in Noc + LatB-treated embryos were unequal with one daughter cell having a much smaller apical cell surface than the other (Figure 7C). In some cases the smaller daughter cells were lost completely from the epithelial layer (Figure 7C). As a consequence, cell division in the Noc + LatB epithelium actually resulted in a net loss of apical cell area (64% ± 3% of predivision surface area) compared to the net gain seen in controls (111% ± 4% of predivision surface area). These embryos also show a thickening of the blastocoel roof (data not shown) and arrest development during epiboly. We suggest that the loss of cells from the epithelium is at odds with the tissue spreading, which must take place at this stage in the embryo, and indicates the importance of keeping a tight control over spindle movement in a proliferating tissue.

## Discussion

The orientation of cell division is a key process that underlies epithelial morphogenesis. In polarized epithelia, the spindle can be oriented either parallel or perpendicular to the plane of the epithelium, resulting in symmetric or asymmetric cell divisions. Symmetric cell divisions underlie tissue spreading or directional tissue morphogenesis, depending on whether the spindle is randomly oriented within the epithelial plane (z axis) or assumes a fixed planar orientation. Fixed planar orientations are observed during fish and frog neurulation controlled by the PCP pathway or Cdc42, respectively (Kieserman and Wallingford, 2009; Quesada-Hernández et al., 2010). Here, we have used the epithelium of the early frog gastrula as a model system to study the least-understood mechanism of symmetric cell divisions, when the spindle is held parallel to the plane of the epithelium but does not assume a fixed orientation within that plane. Such divisions would be important in cases where the epithelium spreads in all directions. The frog gastrula provides a good model system for this because during gastrulation it undergoes epiboly, where the epithelium spreads from the animal to the vegetal pole to cover the entire embryo.

We have shown here that in the early stages of epiboly, epithelial cells in the animal pole divide symmetrically. Spindles exhibit rapid rotation in the x/y axis until anaphase and settle in a variable direction within the epithelial plane, consistent with the requirement of the epithelium to spread in all directions. From a mechanistic point of view, we found that the spindles are positioned by balancing counteracting forces contributed by microtubules/Myo10 on the apical side and actin/myosin-2 on the basal side (Figure S5). In the absence of one or other force, the spindle is repositioned closer to the apical or basal side, respectively, whereas in the absence of both, the spindle is positioning at highly variable points along the apicobasal axis.

Our findings move away from a simple, static, model of spindle positioning whereby spindle location is determined solely by anchoring to a specific cortical landmark, such as an adherens junction (den Elzen et al., 2009; Lu et al., 2001; Marthiens et al., 2010), to a more dynamic system based on antagonistic forces. Interestingly, spindle positioning based on a dynamic balance of forces has also been recently reported for the meiotic spindle in mouse oocytes (Yi et al., 2011) However, our results do not exclude a role for cell-cell junctions in spindle position. One possibility is that they act upstream of the force balancing mechanism we describe here, perhaps by providing the polarity cues necessary to set up such a mechanism. Furthermore, it is also possible that cell junctions assume increased functional importance in mature tissue, rather than in early embryonic epithelia or in cells cultured on artificial substrates.

We find that ablation of either microtubules or Myo10 causes the spindle to reposition apically, suggesting that microtubules and Myo10 provide a basally directed force to position the spindle. What mechanism can explain this? We think clues come from studying the organization of microtubules in these cells; we observe an enrichment of microtubules on the apical side of the cell. It could be that this dense microtubule network functions simply as an apical barrier, preventing the spindle from approaching the apical surface. Alternatively, and/or in addition to a barrier function, this network may exert a pushing force to actively position the spindle. Although we cannot conclusively distinguish between these two possibilities at present, our findings that spindles reposition basally in LatB treatment, MHC-B morphants, and with expression of full-length Myo10 and the microtubule-binding IQT-Myo10 strongly suggest that the microtubule network does exert a basally directed force, in addition to any barrier function. This apical microtubule network is likely to be dynamic because its organization is altered by Myo10 and MHC-B knockdown. It would be interesting to investigate how the dynamic assembly and disassembly of microtubules, which generate forces in other systems (reviewed in Dogterom et al., 2005), contribute to the properties of this network.

Our studies show that Myo10 is required for the formation/maintenance of the apical microtubule network, with knockdown of Myo10 causing a reduction in the apical enrichment of microtubules, which is restored, along with spindle position, when rescued with full-length Myo10. Moreover, we find that the microtubule-binding tail of Myo10 is vital for spindle positioning because the GFP-IQT construct can rescue the morpholino phenotype. In some studies of Myo10 function, the GFP-IQT construct has been shown to act as a dominant negative, presumably because it can still dimerize but cannot function as an actin motor because it lacks the head domain (Cox et al., 2002; Zhang et al., 2004). This is not the case for Myo10's function in spindle position because expression of GFP-IQT in a control background has the opposite effect on spindle position to the Myo10 MO (basal rather than apical position), and the GFP-IQT rescue of the morphant phenotype restores rather than randomizes spindle position. Together, we believe that these results reflect the microtubule rather than actin dependence of Myo10 function in spindle position. Indeed, in spindle position, Myo10 actually functions antagonistically to actin, a role that is consistent with Myo10's function in spindle structure, where F-actin and Myo10 work antagonistically to maintain mitotic spindle length (Woolner et al., 2008). Thus, Myo10 plays crucial roles in the organization of the mitotic spindle, at several levels. A key challenge for the future will be to determine exactly how Myo10 fulfills these functions.

Our photoactivation and pharmacological perturbation experiments demonstrate that the apically directed spindle positioning force depends on actomyosin contraction. Our results are consistent with a model whereby apical actomyosin contraction provides an apically directed force to position the spindle, similar to that which occurs during spindle positioning in oocytes (Schuh and Ellenberg, 2008). However, we also see an apically directed flow of actin filaments, which is dependent on myosin-2 activity. This presents the possibility of a second, nonmutually exclusive model, whereby the spindle is linked to this flow and carried apically.

How is the spindle connected to this flow? Astral microtubules are likely to provide the primary means by which the spindle can connect to the moving cortex. However, because we have shown that spindles move apically in Noc-treated embryos, this suggests that a further, microtubule independent, connection exists. Recent work using live imaging has revealed the presence of dynamic actin cables that reach between the cortex and the spindle both in *Xenopus* embryos and mammalian cells (Fink et al., 2011; Mitsushima et al., 2010; Woolner et al., 2008). These cables could provide a possible link between the spindle and the flowing cortex, even when astral microtubules are lost, and will therefore be an important avenue for future investigation.

The observation that actin and myosin-2 function together to position the spindle bears striking similarities to the force-generating role seen for actomyosin during centrosome separation in single cells (Rosenblatt et al., 2004). Overall, our findings highlight the fact that the actin cytoskeleton is an active, force-generating, contributor to spindle positioning. In previous models, cortical F-actin has been thought to provide a passive substrate in which to anchor dynamic microtubules (Kunda and Baum, 2009), whereas our model suggests a much more dynamic role. Together, these findings highlight the importance of studying the role of actin and actin-based motors during mitosis, an area that, historically, has been dominated by the study of microtubules and their motors (Kunda and Baum, 2009; Sandquist et al., 2011).

What advantages might this mechanism of spindle positioning confer to epithelial tissues? First, we suggest that it allows the spindle to maintain flexibility within the epithelial plane during mitosis, such that the spindle settles in different directions during anaphase, whereas at the same time maintaining a parallel orientation. In this model, the flexibility of direction would allow the epithelium to spread in all directions, whereas the parallel orientation would maintain the epithelial organization. Indeed, in the absence of both microtubule and actomyosin forces, the spindle exhibits rapid “tumbling” movements during mitosis and fails to maintain a parallel orientation. As a consequence, the ectoderm is thickened (data not shown), and the apical ectodermal surface is reduced. Second, we speculate that the molecularly distinct nature of the forces, microtubule/Myo10 on the apical side and F-actin/myosin-2 on the basal side, may endow polarized cells with the inherent ability to vary these forces independently. Finally, we suggest that the dynamic nature of this mechanism may offer an advantage in allowing the spindle to respond rapidly to dynamic cues in the local environment, such as changes in tissue tension. This may be particularly important for marrying cell division plane with tissue shaping during rapid morphogenetic events in embryogenesis or wound healing. Our findings provide a framework of dynamic force interactions, within which some of these ideas can be further tested.

## Experimental Procedures

### *Xenopus laevis* Embryos and Microinjection

Female *Xenopus laevis* frogs were preprimed 4–7 days in advance with 50 U of PMSG (Intervet UK) and then primed with 500 U of HCG (Intervet UK) 18 hr before use. Frogs were kept at 16°C after priming and then transferred to room temperature 1× MMR (100 mM NaCl, 2 mM KCl, 1 mM MgCl, and 5 mM HEPES [pH 7.4]) for egg collection. In vitro fertilization and dejellying were performed as described previously (Woolner et al., 2009). Embryos were microinjected at the two- or four-cell stages into all cells, with needle volumes of 5 and 2.5 nl, respectively, using a Picospritzer III (Parker Instrumentation) with embryos submerged in 0.1× MMR plus 5% Ficoll. RNA for microinjections was made as described previously (Sokac et al., 2003) with needle concentrations as follows: 0.25 mg/ml membrane-GFP (Moriyoshi et al., 1996); 0.5 mg/ml GFP-α-tubulin; 0.1 mg/ml cherry-histone2B (Kanda et al., 1998); 0.0625 mg/ml GFP-Utr-CH (Burkel et al., 2007); 1 mg/ml GFP-HIQT, GFP-IQT GFP-HIQCC, or GFP alone (Weber et al., 2004); and 0.5 mg/ml PAGFP-UtrCH (Burkel et al., 2007). Morpholinos were prepared as described previously (Woolner et al., 2008) and microinjected at a needle concentration of 0.5–1 mM into four-cell stage embryos; morpholinos used were Myo10 MO (5′-TATTCCTCCATGTCTCCCTCTGCTC-3′; Gene Tools, LLC), MHC-B MO (5′-CTTCCTGCCCTGGTCTCTGTGACAT-3′) (Skoglund et al., 2008), and standard control MO (5′-CCTCTTACCTCAGTTACAATTTATA-3′).

### Drug Treatments

LatB (Sigma-Aldrich), Noc (Sigma-Aldrich), and Jas (Merck Chemicals) stocks were made up in DMSO and diluted in 0.1× MMR to give final concentrations of 2.5 μM, 50 nM, and 10 μM, respectively, and DMSO concentrations of 0.1% for LatB and Noc and 1% for Jas. Stage 10 embryos were soaked in these drug solutions for 30 min at room temperature (alongside an appropriate DMSO control) and then immediately fixed for immunofluorescence. Y27632 (Sigma-Aldrich) was solubilized in ddH_2_O and microinjected into the blastocoel of stage 10 embryos; two injections of 16 nl were made into opposite sides of the blastocoel with a needle concentration of 15 mM (will give a final concentration in the blastocoel of approximately 1.5 mM). Control embryos were microinjected with the same volume of ddH_2_O. After injection, embryos were incubated at room temperature for 30 min and then fixed immediately.

### Immunofluorescence

Embryos were fixed for immunofluorescence at stage 10–10.5 (approximately 18 hr postfertilization at 16°C) and processed using a modified version of the protocol developed by Danilchik et al. (1998), omitting the methanol postfix and bisecting, quenching and bleaching (methanol-free) in that order. Embryos were incubated in primary and secondary antibodies in TBSN/BSA (Tris-buffered saline: 155 mM NaCl, 10 mM Tris-Cl [pH 7.4]; 0.1% Nonidet P-40; 10 mg/ml BSA) overnight at 4°C, with five 1 hr washes with TBSN/BSA following each incubation. Primary antibodies were used at a dilution of 1:200 and were as follows: anti-α-tubulin (DM1A; Sigma-Aldrich); anti-β-catenin (Abcam); anti-GFP (Invitrogen); anti-phospho-myosin light chain 2 (Ser19) (Cell Signaling Technology); and anti-ZO-1 (Invitrogen). Alexa Fluor secondary antibodies (Invitrogen) were used at a dilution of 1:400. To stain DNA, DAPI (Invitrogen), at a final concentration of 10 μg/ml, was added to one of the final TBSN washes and incubated for 30 min at room temperature. After staining, embryos were dehydrated in methanol and cleared and mounted in Murray's Clear (2:1, benzyl benzoate:benzyl alcohol). A detailed description of the image processing and statistical analysis used in this study can be found in the Supplemental Experimental Procedures.

## Supplemental Information

Document S1. Figures S1–S5 and Supplemental Experimental Procedures

Movie S1. Related to Figure 1. Mitotic Spindle Positioning in Epithelial Cells of *Xenopus laevis* Gastrula Live confocal imaging of mitotic spindles in the outer epithelium of a stage 10 *Xenopus laevis* embryo. GFP-α-tubulin (green) was used to label microtubules, and chromosomes were highlighted using mCherry-Histone2B (red). Images were collected from a single focal plane every 6 s and reconstructed at a speed of 12 frames per second (fps). Note that spindles undergo rapid movements in the x/y axis but remain oriented parallel to the plane of the epithelium and stay positioned in a specific location along the apicobasal axis.

Movie S2. Related to Figure 1. 3D Reconstruction of Nuclei in Embryonic Epithelium Nuclei were reconstructed from a z stack movie of mcherry-Histone2B in an epithelial cell of *Xenopus* embryo using Imaris image analysis software. The reconstruction illustrates that condensed chromosomes undergo very little movement in the apicobasal axis as cell division takes place and two daughter nuclei are formed.

Movie S3. Related to Figure 3. Side-View Movies of Mitotic Spindle in Control and Nocodazole-Treated Embryos Z slice side-view movies of mitotic spindles in a control embryo (left, Ctrl) and in an embryo treated with low-concentration nocodazole (right, Noc) to specifically disrupt astral microtubules. The apical cell surface is at the top of each movie; microtubules are visualized using GFP-α-tubulin (green), and chromosomes are highlighted using mCherry-H2B (red). The right-hand embryo was placed in nocodazole approximately 10 min before the start of the movie. The control spindle undergoes virtually no movement in the z (apicobasal) axis, whereas the Noc-treated spindle moves toward the apical cell surface. Images were collected every 4.3 s and are shown at 7 fps.

Movie S4. Related to Figure 3. Spindles Still Assemble in Low-Concentration Nocodazole A low-resolution movie demonstrating that mitotic spindles (visualized by GFP-α-tubulin) continue to assemble in low-concentration (100 nM) nocodazole. The embryo was placed in nocodazole 22 min before the start of the movie; images were collected every 7 s and are shown at 15 fps.

Movie S5. Related to Figure 6. Apical-ward Movement of F-Actin Seen by Loss of Photoactivation Fluorescence A z slice side-view movie showing PAGFP-UtrCH (green) in a mitotic cell after photoactivation (green oval) of the cell cortex. Once photoactivation is stopped, loss of fluorescence begins basally and spreads in an apical direction, suggestive of an apical-ward movement of F-actin. Images were taken every 793 ms and are shown at 15 fps.

Movie S6. Related to Figure 6. F-Actin Movement in Control and MHC-B MO Embryos A z slice movie of photoactivated PAGFP-UtrCH in a control embryo (left) and MHC-B MO-injected embryo (right). These movies were used to make the kymographs in Figures 6H and 6I, respectively. In the control, fluorescence is lost first from the basal side and spreads apically. When myosin-2 is knocked down using MHC-B MO, fluorescence loss no longer starts basally but is random and nondirectional. Images were taken every 793 ms and are shown at 8 fps.

Movie S7. Related to Figure 7. Spindles in Noc + LatB “Tumble” out of Plane Live imaging of spindles (green; GFP-α-tubulin) and chromosomes (red; Cherry-H2B) in an embryo incubated in Noc + LatB from 15 min before the start of the movie. As the movie progresses, spindles can be seen rocking out of the plane of the epithelium, unlike control spindles, which always stay level in the plane (see Movie S1). This results in unequal cell divisions in which daughter cells have differing apical surface areas. In some instances, daughter cells with a small apical surface are lost from the epithelial layer. Images were taken every 7 s and are shown at 12 fps.
