# Supplementary material for: Spindle Position in Symmetric Cell Divisions during Epiboly Is Controlled by Opposing and Dynamic Apicobasal Forces
Source: Dev Cell. 2012 Apr 17;22(4):775–87. doi: 10.1016/j.devcel.2012.01.002 (PMC3332010; doi:10.1016/j.devcel.2012.01.002)
Supplement: Document S1. Figures S1–S5 and Supplemental Experimental Procedures [file mmc1.pdf]

Developmental Cell, Volume 22

Supplemental Information

Spindle Position in Symmetric Cell Divisions during Epiboly Is Controlled by Opposing and Dynamic Apicobasal Forces

Sarah Woolner and Nancy Papalopulu

INVENTORY OF SUPPLEMENTAL INFORMATION

Figure S1, related to Figure 3. Images and quantification of spindle position in embryos treated with increasing concentrations or increasing temporal exposures to nocodazole.

Figure S2, related to Figure 4. Quantification of interphase nuclei position and metaphase spindle length in nocodazole and latrunculin B treatments.

Figure S3, related to Figure 5. Quantification of nuclei position in myosin-10 morphants, images showing the localisation of full-length myosin-10, quantification of spindle position following overexpression of myosin-10 truncation constructs and quantification of metaphase spindle length in myosin-10 morphants.

Figure S4, related to Figure 6. Quantification of interphase nuclei position and metaphase spindle length in MHC-B morphants. Reconstructions of microtubule organisation in control and MHC-B morphants.

Figure S5, related to Figure 7. A schematic diagram detailing our working model for spindle positioning via antagonistic microtubule and actomyosin forces.

Supplemental Experimental Procedures

Confocal image collection and processing, a description of the procedures used to collect and process confocal movies and images.

Quantification and statistical analysis, a description of the methods and statistical tests used to quantify apicobasal spindle position, nucleus position, spindle length and spindle angle.

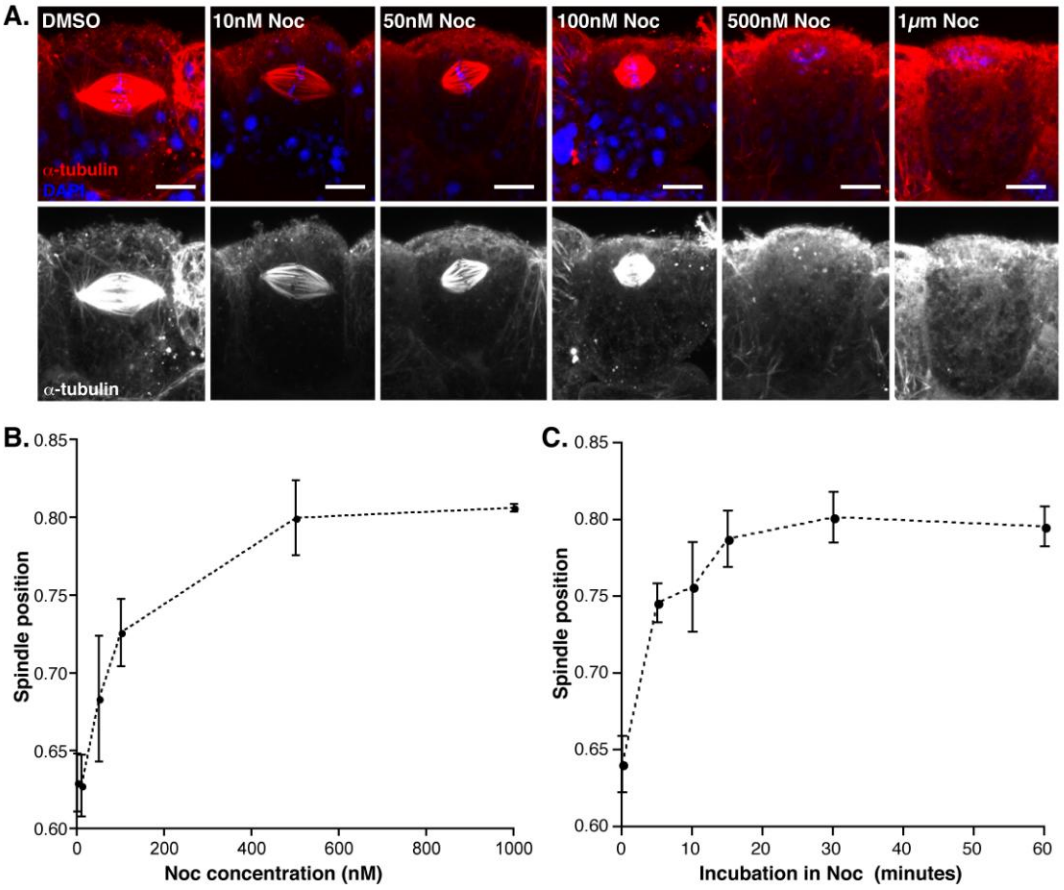

Figure S1, related to Figure 3: (A) Confocal images from embryos incubated in a concentration gradient of nocodazole (Noc) from 10nM to 1µM. Spindles reposition more apically as Noc concentration increases and microtubules are lost. In 500nM and 1µM Noc, spindle microtubules are lost and condensed chromosomes are found pressed against the apical cell surface. Scale bars represent 10µm. **(B)** Quantification of spindle position in Noc concentration gradient (error bars represent standard error of the mean, n = 3 independent experiments from a total of 13-18 embryos for each treatment). **(C)** Quantification of spindle position in a time series experiment, where embryos were incubated in 100nM Noc for varying time periods, from 0 to 60 mins. The average spindle position remained apical in longer incubations, indicating that spindles assembled after the addition of Noc, as well as existing spindles, take up the apical position (error bars represent standard error of the mean, n = 3 independent experiments from a total of 10-18 embryos for each treatment).

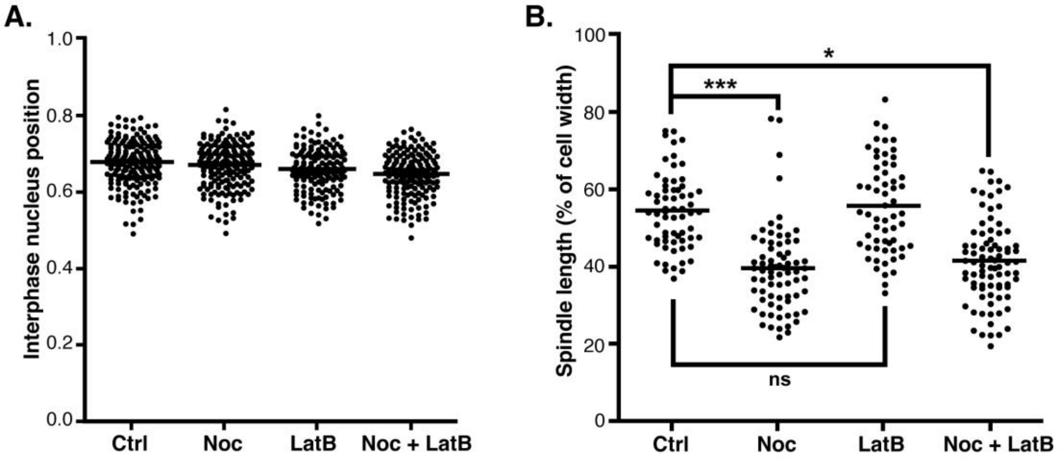

Figure S2, related to Figure 4: (A) Interphase nuclei position was quantified in Control, Noc, LatB and Noc + LatB treatments. Position was measured relative to cell length. No significant difference in interphase nuclei position was see in any of the treatments. (**B)** Metaphase spindle length was measured in Control, Noc, LatB and Noc + LatB treatments. Spindle length was measured relative to cell width. Spindles in embryos treated with Noc were found to be significantly shorter when compared to controls. To test for significance, unpaired Student's t-tests were performed (n=3 independent experiments from a total of 18, 18, 16, 17, embryos for Ctrl, Noc, LatB and Noc + LatB respectively; *p<0.05, ***p<0.001).

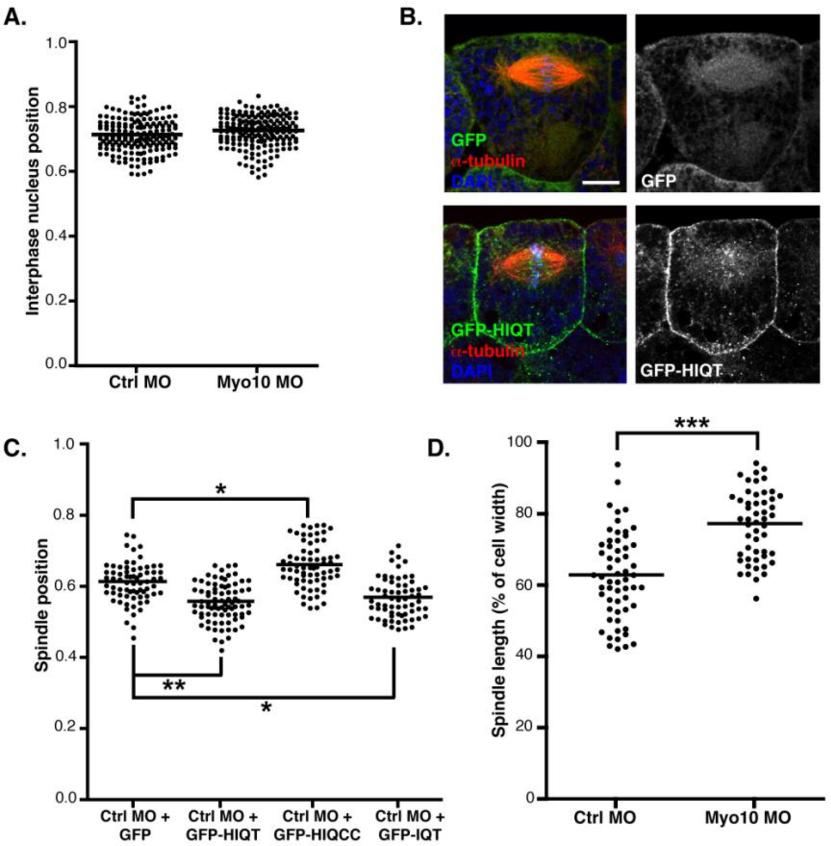

Figure S3, related to Figure 5: (A) Quantification of interphase nuclei position in Control MO and Myo10 MO injected embryos. Nuclei position was measured relative to cell length. No significant difference in nuclei position was seen (an unpaired Student's t-test was performed; n=3 independent experiments from a total of 21 embryos for Ctrl MO and Myo10 MO). **(B)** To determine Myo10 localisation, embryos were injected with GFP or GFP-HIQT (full length Myo10 tagged with GFP) and stained using an anti-GFP antibody (green). GFP-HIQT localises to the spindle and shows strong accumulation at the cell cortex. **(C)** Quantification of spindle position following over-expression of Myo10 constructs. In a control background, expression of GFP-HIQT causes spindles to reposition slightly basally, as does expression of GFP-IQT. Whereas, expression of GFP-HIQCC causes spindles to reposition slightly apically when compared to controls. To test for significance, unpaired Student's t-tests were performed (n=3 independent experiments, from a total of 20, 19, 18, and 16 embryos each for Ctrl MO + GFP, + GFP-HIQT + GFP-HIQCC and + GFP-IQT, respectively; *p<0.05, **p<0.01). **(D)** Quantification of metaphase spindle length in Ctrl and Myo10 MO injected embryos, measured relative to cell width. Spindles in Myo10 morphants are significantly longer than controls (an unpaired Student's t-test was performed; n=3 independent experiments from a total of 21 embryos for Ctrl MO and Myo10 MO, ***p<0.001).

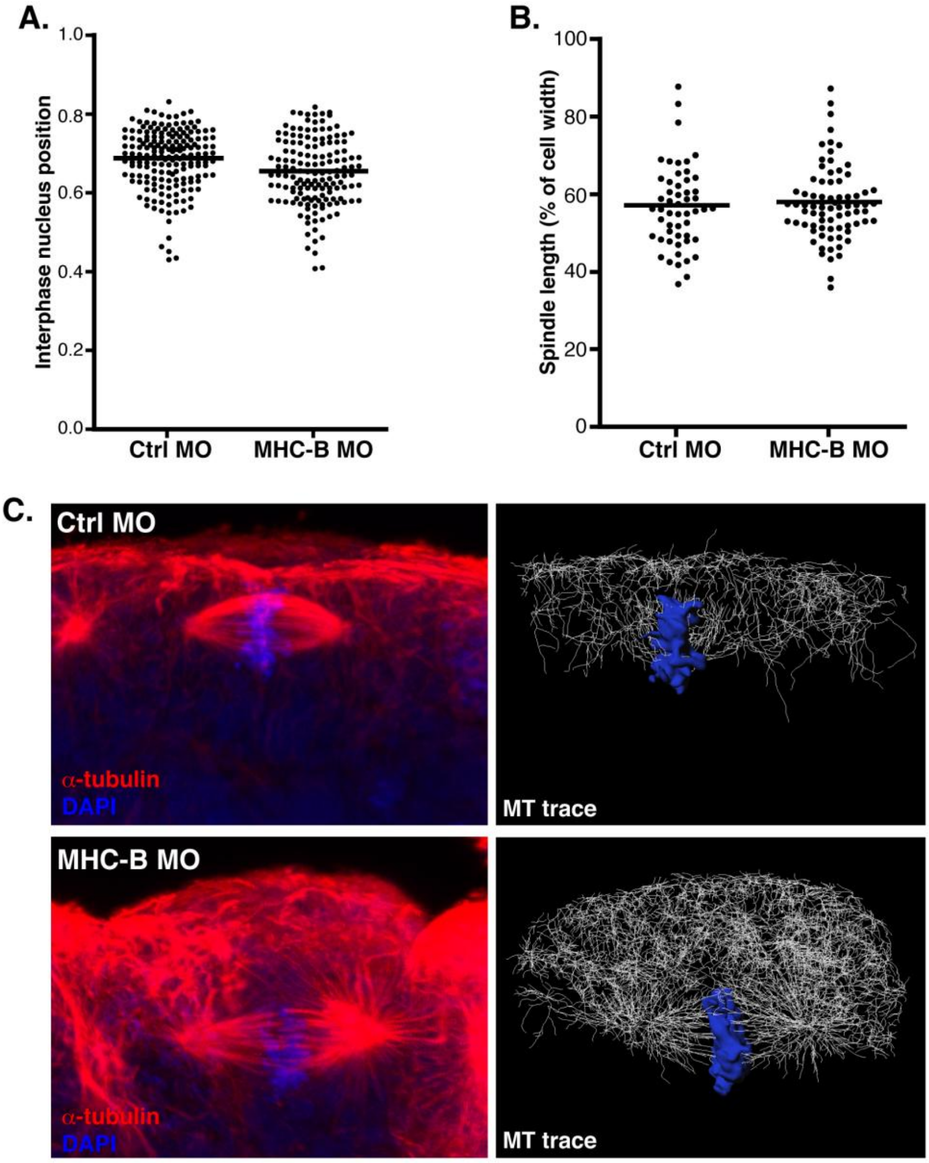

Figure S4, related to Figure 6: (A) Quantification of interphase nuclei position in Control MO and MHC-B MO injected embryos. Nuclei position was measured relative to cell length. No significant difference in nuclei position was seen. **(B)** Quantification of metaphase spindle length in Ctrl and MHC-B MO injected embryos, measured relative to cell width. No significant difference in spindle length was seen. **(C)** Microtubule organisation following knockdown of myosin-2. Z-stack (left; α-tubulin in red, DAPI staining in blue) and microtubule trace (right; trace in white; chromosomes reconstructed in blue) images of Control and MHC-B MO cells. Knockdown of myosin-2 leads to an expansion of the dense microtubule network apical of the spindle. To test for statistical significance in (A) and (B), unpaired Student's t-tests were performed (n=3 independent experiments from a total of 21 and 25 embryos for Ctrl MO and MHC-B MO, respectively).

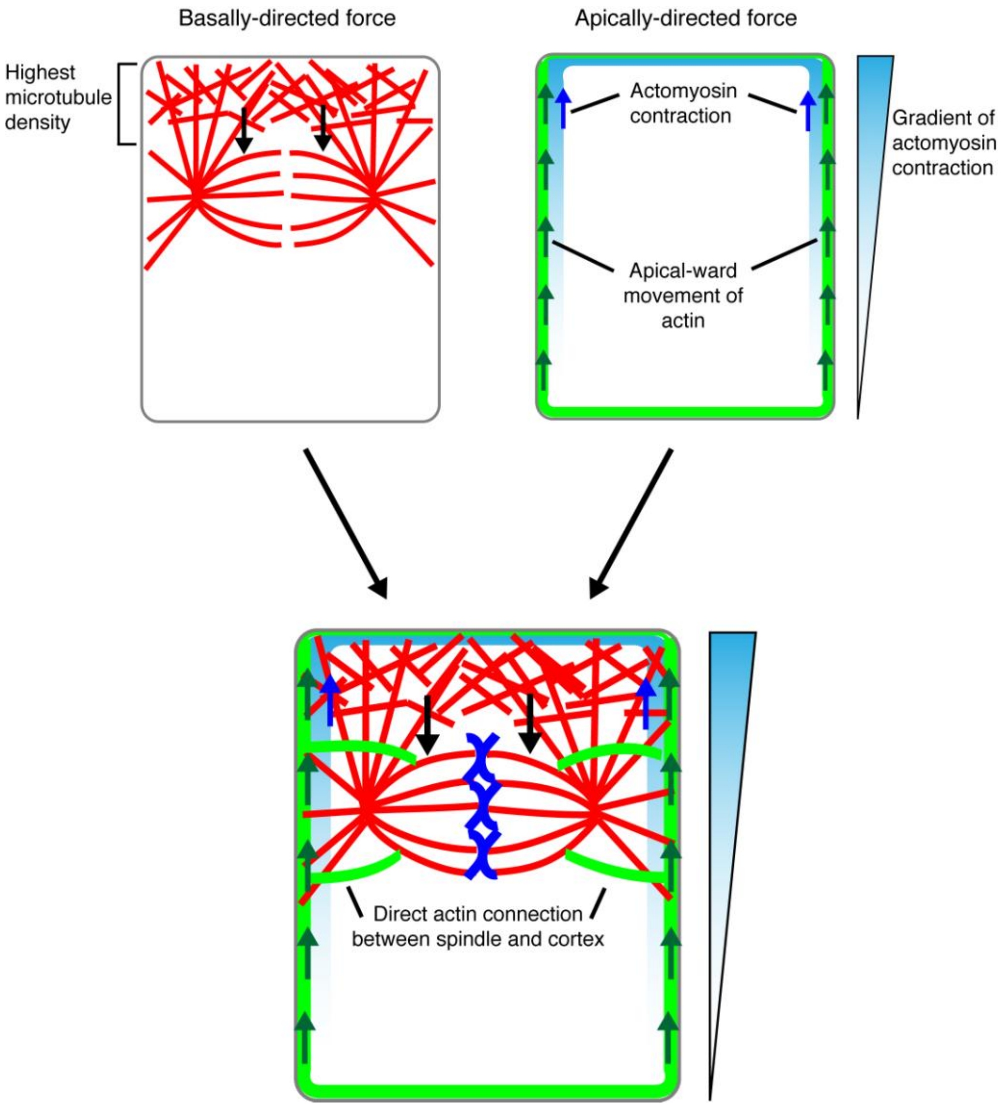

Figure S5, related to Figure 7: A working model for spindle positioning during symmetric divisions. Our results indicate that spindle position during symmetric division is determined by a balance between a basally-directed force (left) and an apically-directed force (right). Microtubule organisation shows an apical bias with astral microtubules contributing to a dense microtubule network apical of the spindle. This forms an apical barrier, preventing the spindle from moving apically and pushing the spindle basally. Active myosin-2 is concentrated in the apical portion of the cell, resulting in a gradient of actomyosin contraction from apical to basal (graded blue triangle). Apical contraction may help pull the spindle apically but will also generate an apical-ward movement of cortical F-actin. The spindle is connected to flowing cortical F-actin via astral microtubules and actin cables, potentially carrying the spindle apically.

SUPPLEMENTAL EXPERIMENTAL PROCEDURES

Confocal image collection and processing

All confocal imaging was performed on a Leica SP5 II confocal microscope with z-galvo stage. For side-view images of fixed spindles, sagittally bisected embryos were imaged by mounting with the cut side against the coverslip. Live imaging was performed as described previously (Woolner et al., 2009). For side-view images of live spindles, xzt movies were collected using the z-galvo stage. Photoactivation experiments were carried out using xzt imaging, with a region of interest drawn to define a zone in which light from the 405nm laser was used to activate the PA-GFP probe. To avoid artifacts with photoactivating from the apical side, we followed the loss of fluorescence after cessation of activation with the 405nm. Confocal images and movies were processed using Image J and Imaris (Bitplane Scientific Software) image analysis software. For 3D reconstruction of fixed spindles, z-stack images were collected by imaging from the apical surface of the embryo and then processed using Imaris. Spindles and DNA were rendered with Imaris using the surface reconstruction tool. Single cells from the epithelial sheet were reconstructed with Imaris using the contour surface tool. To trace microtubules in an unbiased manner, the automatic filament trace tool in Imaris was applied to side-view z-stack images, using the same starting parameters for each sample (largest diameter: 1µm; thinnest diameter: 0.1µm). Kymographs of F-actin movement were generated using Adobe Photoshop CS3, by batch selecting the same region on all images of a photoactivation movie and adjusting slightly in the case of any movement of the cortex during the movie. Regions were then cropped and pasted in chronological order to produce a kymograph.

Quantification and statistical analysis

To measure spindle position, low magnification (40x objective) side-view images of fixed embryos were collected and analysed with Imaris using the line measurement tool. To control for differences in cell size, we calculated a ratio of spindle distance from the apical surface against cell length (1-[distance from apical cell surface to spindle center/distance from apical to basal cell surface]), such that a spindle position of 1 is the most apical and a position of 0 is the most basal. Interphase nucleus position was quantified in a similar fashion, measuring to the center of each nucleus. For spindle length measurements, pole-to-pole distance in metaphase spindles was measured using side-view z-stacks. To control for differences in cell size, spindle length was calculated as a percentage of cell width. To test for statistical significance, the mean spindle positions for each experiment were calculated and unpaired Student t-tests were performed. For spindle angle measurements, z-stack images taken from the apical surface of the epithelium were analysed in Image J (NIH) and spindle angles were measured using a 3D spindle angle macro (a gift from Jeremy Green, KCL) (Tabler et al., 2010).
