# Supplementary figures and images for: Spindle Position in Symmetric Cell Divisions during Epiboly Is Controlled by Opposing and Dynamic Apicobasal Forces
Source: Dev Cell. 2012 Apr 17;22(4):775–87. doi: 10.1016/j.devcel.2012.01.002 (PMC3332010; doi:10.1016/j.devcel.2012.01.002)

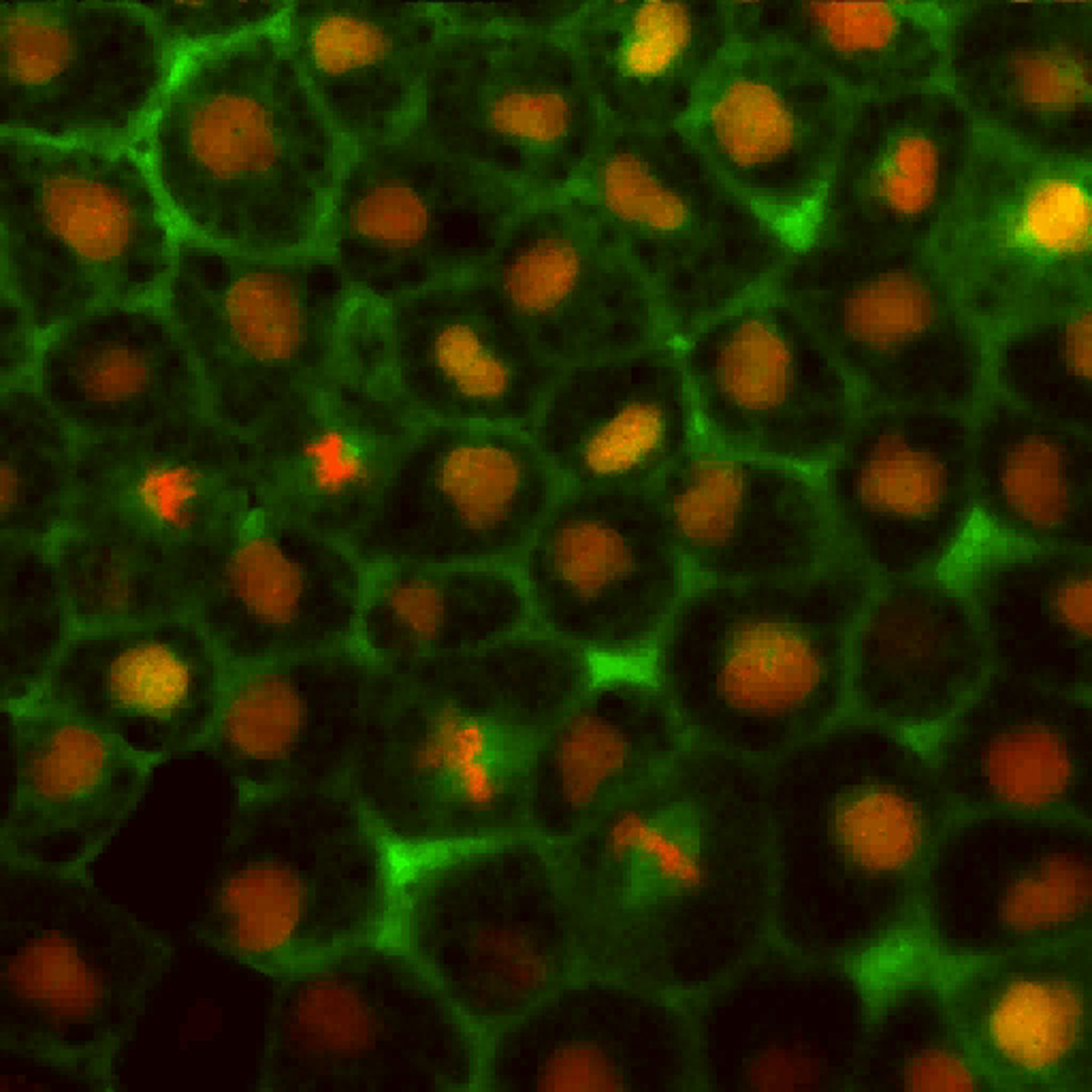

Supplement: Movie S1. Related to Figure 1. Mitotic Spindle Positioning in Epithelial Cells of Xenopus laevis Gastrula — Live confocal imaging of mitotic spindles in the outer epithelium of a stage 10 Xenopus laevis embryo. GFP-α-tubulin (green) was used to label microtubules, and chromosomes were highlighted using mCherry-Histone2B (red). Images were collected from a single focal plane every 6 s and reconstructed at a speed of 12 frames per second (fps). Note that spindles undergo rapid movements in the x/y axis but remain oriented parallel to the plane of the epithelium and stay positioned in a specific location along the apicobasal axis. [file mmc2.jpg]

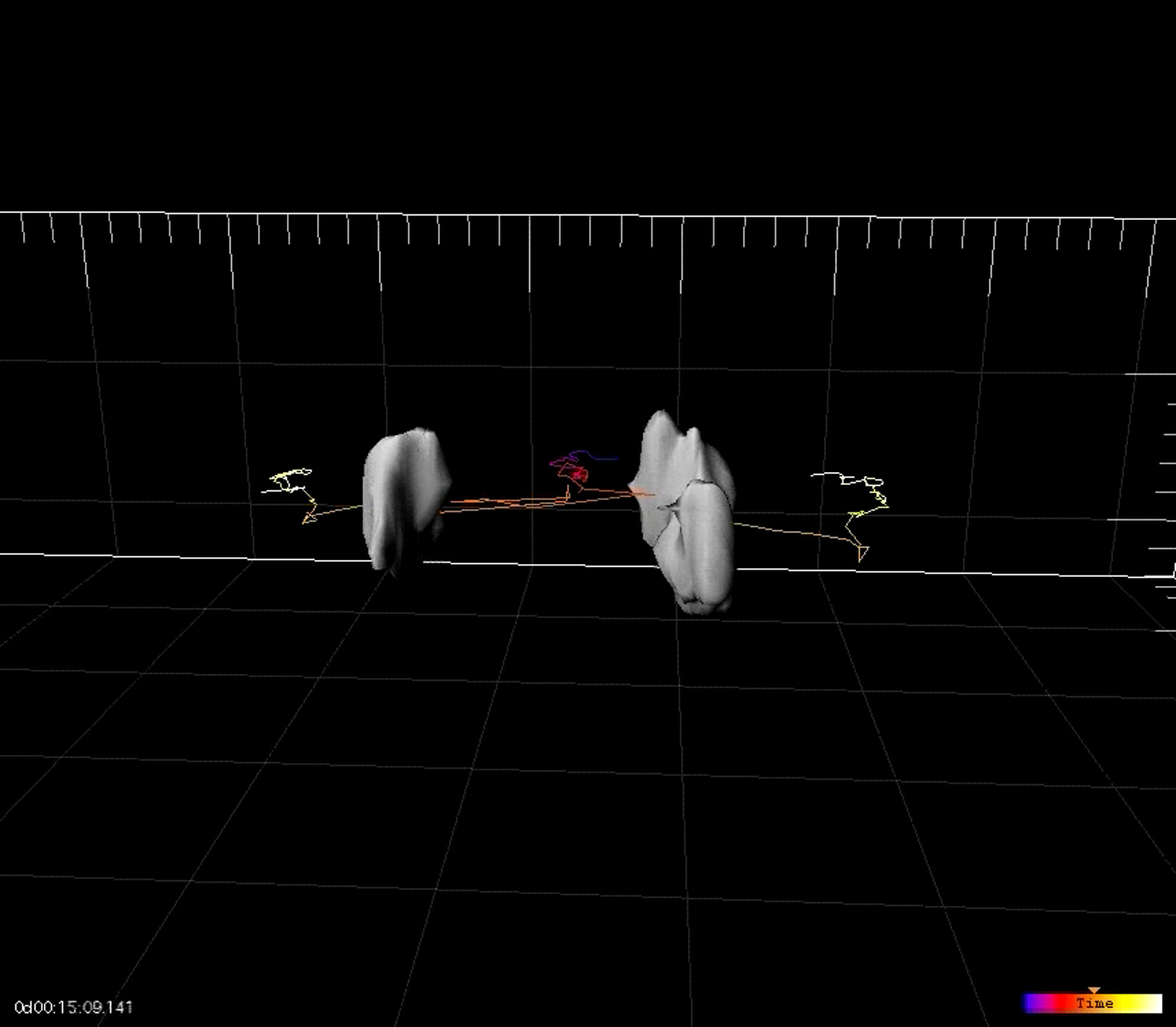

Supplement: Movie S2. Related to Figure 1. 3D Reconstruction of Nuclei in Embryonic Epithelium — Nuclei were reconstructed from a z stack movie of mcherry-Histone2B in an epithelial cell of Xenopus embryo using Imaris image analysis software. The reconstruction illustrates that condensed chromosomes undergo very little movement in the apicobasal axis as cell division takes place and two daughter nuclei are formed. [file mmc3.jpg]

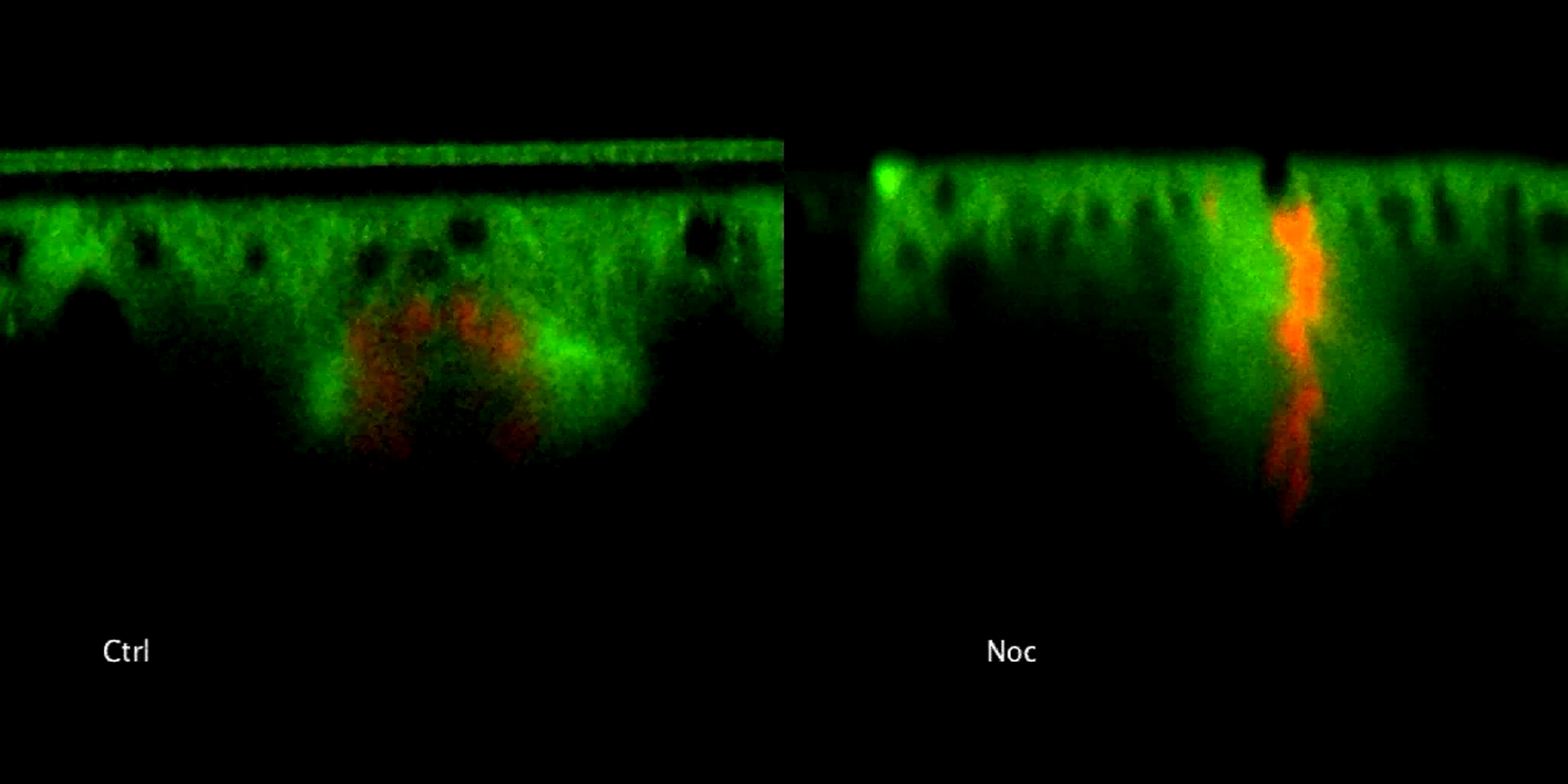

Supplement: Movie S3. Related to Figure 3. Side-View Movies of Mitotic Spindle in Control and Nocodazole-Treated Embryos — Z slice side-view movies of mitotic spindles in a control embryo (left, Ctrl) and in an embryo treated with low-concentration nocodazole (right, Noc) to specifically disrupt astral microtubules. The apical cell surface is at the top of each movie; microtubules are visualized using GFP-α-tubulin (green), and chromosomes are highlighted using mCherry-H2B (red). The right-hand embryo was placed in nocodazole approximately 10 min before the start of the movie. The control spindle undergoes virtually no movement in the z (apicobasal) axis, whereas the Noc-treated spindle moves toward the apical cell surface. Images were collected every 4.3 s and are shown at 7 fps. [file mmc4.jpg]

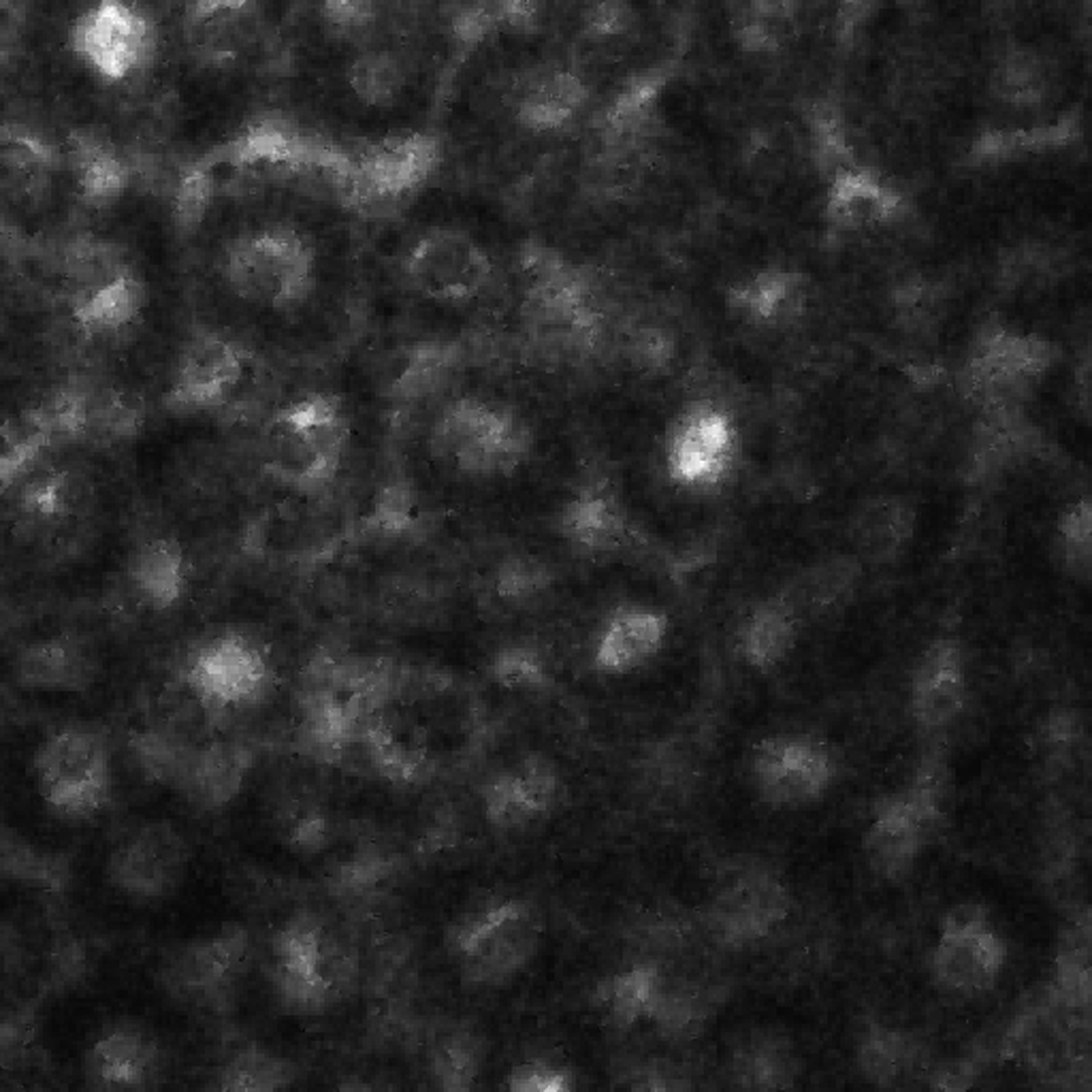

Supplement: Movie S4. Related to Figure 3. Spindles Still Assemble in Low-Concentration Nocodazole — A low-resolution movie demonstrating that mitotic spindles (visualized by GFP-α-tubulin) continue to assemble in low-concentration (100 nM) nocodazole. The embryo was placed in nocodazole 22 min before the start of the movie; images were collected every 7 s and are shown at 15 fps. [file mmc5.jpg]

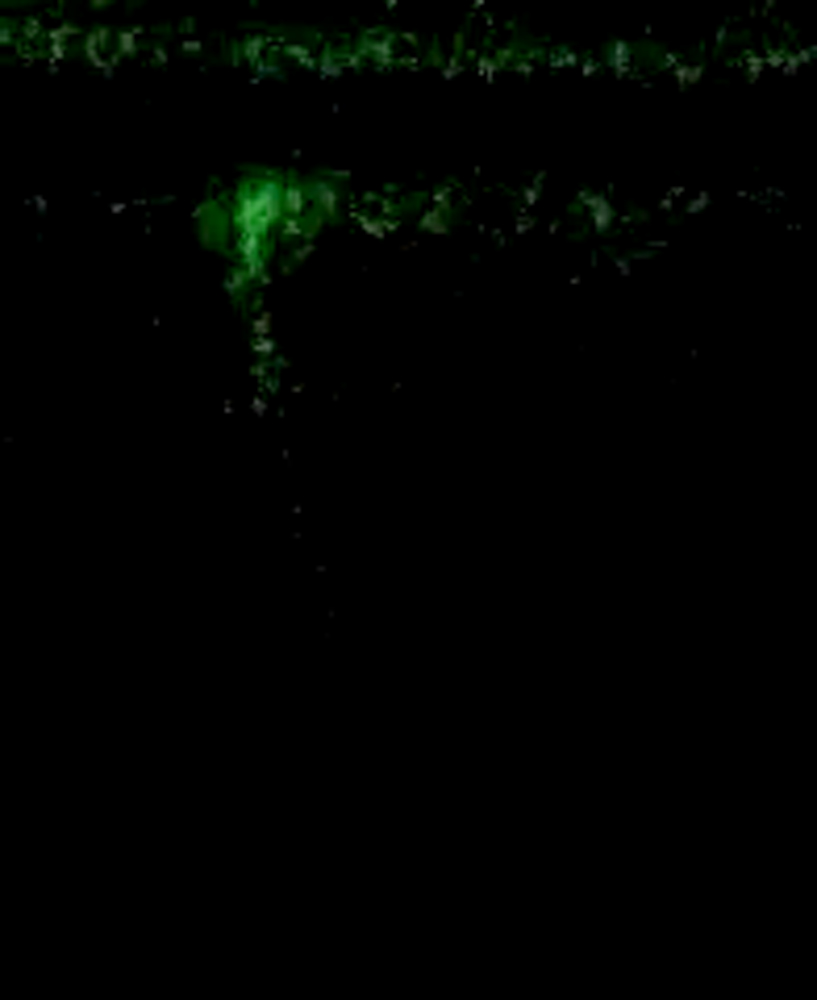

Supplement: Movie S5. Related to Figure 6. Apical-ward Movement of F-Actin Seen by Loss of Photoactivation Fluorescence — A z slice side-view movie showing PAGFP-UtrCH (green) in a mitotic cell after photoactivation (green oval) of the cell cortex. Once photoactivation is stopped, loss of fluorescence begins basally and spreads in an apical direction, suggestive of an apical-ward movement of F-actin. Images were taken every 793 ms and are shown at 15 fps. [file mmc6.jpg]

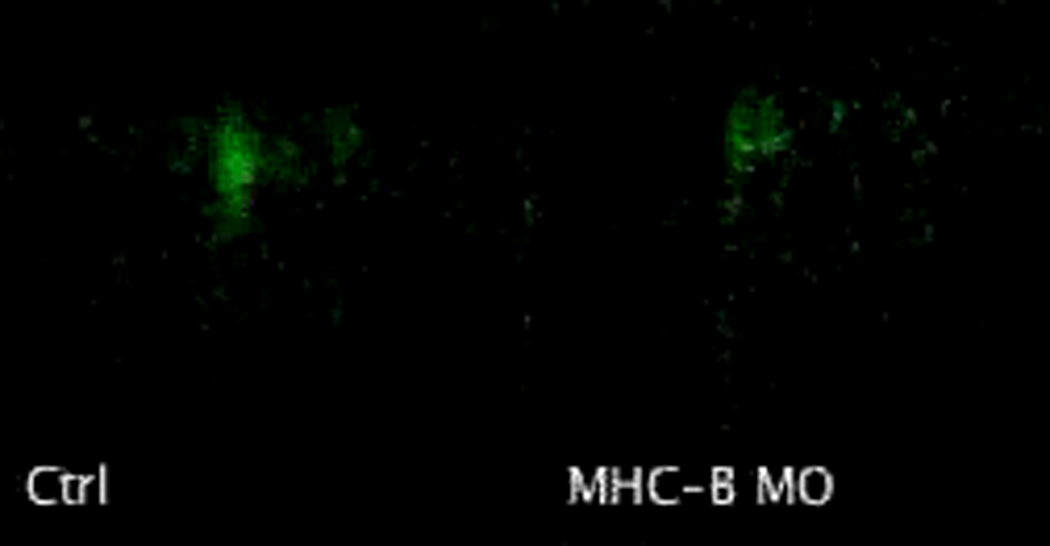

Supplement: Movie S6. Related to Figure 6. F-Actin Movement in Control and MHC-B MO Embryos — A z slice movie of photoactivated PAGFP-UtrCH in a control embryo (left) and MHC-B MO-injected embryo (right). These movies were used to make the kymographs in Figures 6H and 6I, respectively. In the control, fluorescence is lost first from the basal side and spreads apically. When myosin-2 is knocked down using MHC-B MO, fluorescence loss no longer starts basally but is random and nondirectional. Images were taken every 793 ms and are shown at 8 fps. [file mmc7.jpg]

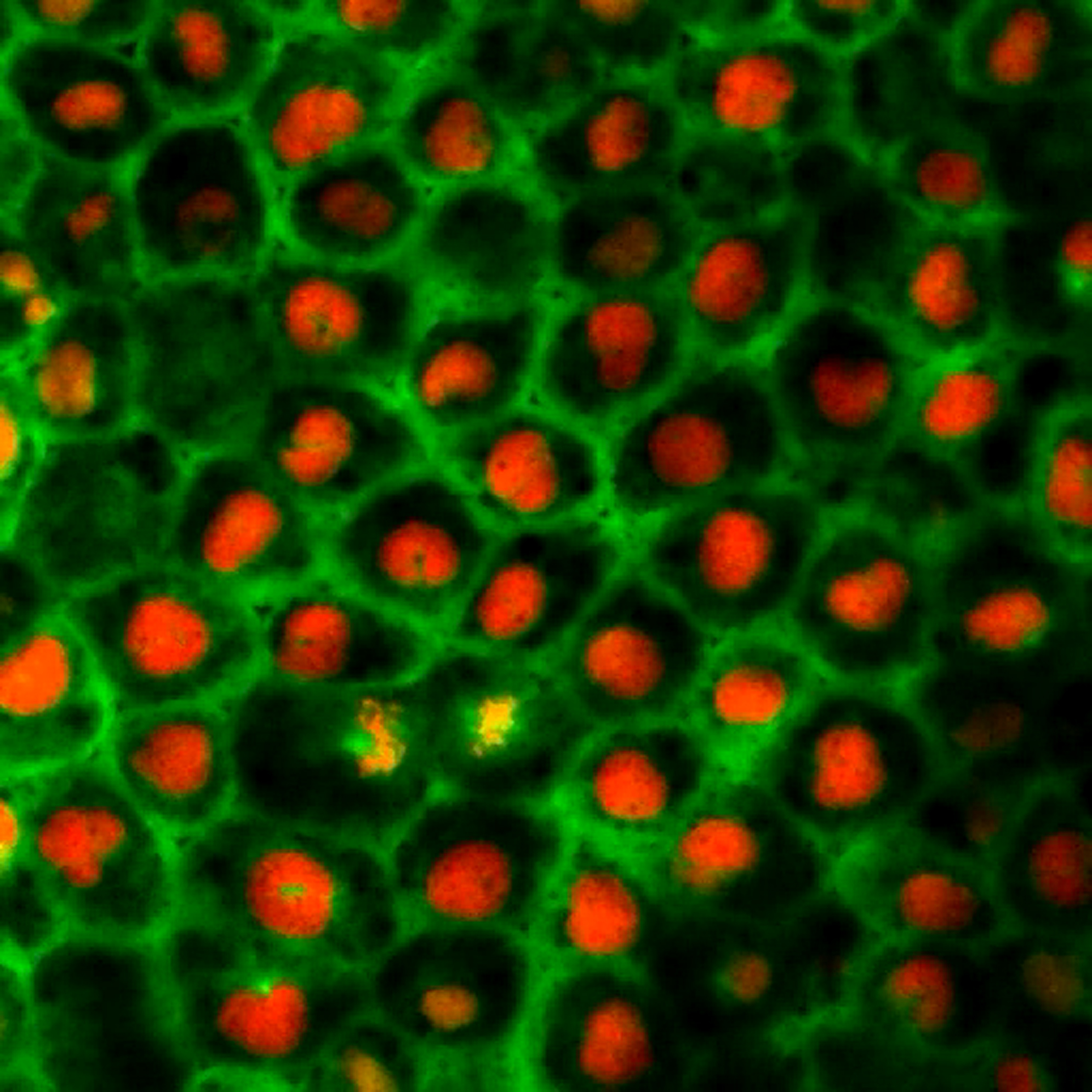

Supplement: Movie S7. Related to Figure 7. Spindles in Noc + LatB “Tumble” out of Plane — Live imaging of spindles (green; GFP-α-tubulin) and chromosomes (red; Cherry-H2B) in an embryo incubated in Noc + LatB from 15 min before the start of the movie. As the movie progresses, spindles can be seen rocking out of the plane of the epithelium, unlike control spindles, which always stay level in the plane (see Movie S1). This results in unequal cell divisions in which daughter cells have differing apical surface areas. In some instances, daughter cells with a small apical surface are lost from the epithelial layer. Images were taken every 7 s and are shown at 12 fps. [file mmc8.jpg]
